# Mutations in *fam20b* and *xylt1* Reveal That Cartilage Matrix Controls Timing of Endochondral Ossification by Inhibiting Chondrocyte Maturation

**DOI:** 10.1371/journal.pgen.1002246

**Published:** 2011-08-25

**Authors:** B. Frank Eames, Yi-Lin Yan, Mary E. Swartz, Daniel S. Levic, Ela W. Knapik, John H. Postlethwait, Charles B. Kimmel

**Affiliations:** 1 Institute of Neuroscience, University of Oregon, Eugene, Oregon, United States of America; 2 Department of Medicine and Department of Cell and Developmental Biology, Vanderbilt University, Nashville, Tennessee, United States of America; University of Oxford, United Kingdom

## Abstract

Differentiating cells interact with their extracellular environment over time. Chondrocytes embed themselves in a proteoglycan (PG)-rich matrix, then undergo a developmental transition, termed “maturation,” when they express *ihh* to induce bone in the overlying tissue, the perichondrium. Here, we ask whether PGs regulate interactions between chondrocytes and perichondrium, using zebrafish mutants to reveal that cartilage PGs inhibit chondrocyte maturation, which ultimately dictates the timing of perichondral bone development. In a mutagenesis screen, we isolated a class of mutants with decreased cartilage matrix and increased perichondral bone. Positional cloning identified lesions in two genes, *fam20b* and *xylosyltransferase1* (*xylt1*), both of which encode PG synthesis enzymes. Mutants failed to produce wild-type levels of chondroitin sulfate PGs, which are normally abundant in cartilage matrix, and initiated perichondral bone formation earlier than their wild-type siblings. Primary chondrocyte defects might induce the bone phenotype secondarily, because mutant chondrocytes precociously initiated maturation, showing increased and early expression of such markers as *runx2b*, *collagen type 10a1*, and *ihh* co-orthologs, and *ihha* mutation suppressed early perichondral bone in PG mutants. Ultrastructural analyses demonstrated aberrant matrix organization and also early cellular features of chondrocyte hypertrophy in mutants. Refining previous *in vitro* reports, which demonstrated that *fam20b* and *xylt1* were involved in PG synthesis, our *in vivo* analyses reveal that these genes function in cartilage matrix production and ultimately regulate the timing of skeletal development.

## Author Summary

The formation of bone around a cartilage template is the predominant ossification process in humans, and it has many complex steps that must be coordinated properly in space and time. Cartilage-producing cells (chondrocytes) first secrete a cartilage matrix around themselves that is rich in sugar-coated proteins called proteoglycans (PGs). Chondrocytes then undergo a maturation process during which they express Indian hedgehog (Ihh), a secreted protein that stimulates bone formation in surrounding cells. Here, we find that PGs in cartilage matrix regulate the timing of chondrocyte maturation, showing that interactions between cells and an environment that they themselves create are crucial for proper skeletal development. Our conclusions are based upon analyses of zebrafish with mutations in two different PG synthesis genes that produce identical skeletal defects. We argue that, as a consequence of deficient cartilage PG production, mutant chondrocytes accelerate the timing of *ihh* expression, which we show causes early and increased bone production in the surrounding cells.

## Introduction

Vertebrate bone is produced by two major developmental processes, intramembranous ossification (forming dermal bones) and endochondral ossification (forming chondral bones). The latter process has many stages that must be coordinated in space and time. First, some cells in the mesenchymal condensation differentiate as chondrocytes, which secrete a cartilage extracellular matrix rich in proteoglycans (PGs), such as chondroitin sulfate PGs [1], [2]. A thin layer of cells, the perichondrium, surrounds the developing cartilage. Later, a subset of chondrocytes undergoes a developmental transition, expressing markers of chondrocyte maturation, including *indian hedgehog (ihh)* and *collagen type 10a1* (*col10*; [3]). Meanwhile, cells of the perichondrium that overlie the maturing chondrocytes differentiate as bone-forming osteoblasts.

Tissue interactions between cartilage and perichondrium ensure that these early events in endochondral ossification are coordinated in space and time [4]–[6]. Critically, the growth factor Ihh produced by maturing chondrocytes induces perichondral bone, and both mouse *Ihh* and zebrafish *ihha* mutants have delayed perichondral ossification [7], [8]. Although specific signaling pathways and transcription factors are involved [9]–[11], the full ontology of genes that regulate the timing of endochondral ossification is still unknown, but may include genes involved in extracellular matrix production.

Mutations disrupting PG synthesis commonly affect skeletal tissues and can change the timing of skeletal development. Hereditary Multiple Exostoses is caused by mutations in *Exostosin (Ext)* genes, which encode enzymes that synthesize heparan sulfate PGs [12], [13]. Mouse and zebrafish models of *Ext* loss-of-function reveal defects in endochondral ossification associated with delays in chondrocyte maturation, which were attributed to altered Ihh signaling [14]–[16]. Mutations affecting various components of PG synthesis, from sugar precursor production enzymes to sulfation enzymes, affect dramatically the shape and composition of skeletal tissues, and also can delay endochondral ossification [14], [17]–[20]. Interestingly, acceleration or inhibition of developmental timing may be a function of the class of PG. Evidence above indicates that mutations in HSPG synthesis delay endochondral ossification, while mutations in chondroitin sulfate PG (CSPG) synthesis may accelerate this bone-forming process. Aggrecan is the major chondroitin sulfate PG (CSPG) in cartilage, and *Aggrecan (Acan)* mutant chicks exhibit increased levels of *Col10* expression [21], which these authors interpreted as evidence that endochondral ossification had initiated earlier. Here we perform direct investigation of developmental timing in order to test the hypothesis that loss of CSPGs accelerates endochondral ossification.

In a forward genetic screen of skeletal tissues in developing zebrafish larvae, we isolated a class of mutants with increased chondral bone and decreased cartilage matrix at 6 days post-fertilization (dpf). Positional cloning identified mutations in *fam20b* and *xylosyltransferase1 (xylt1)*, two critical genes for PG synthesis that are associated with human disease [22]–[27]. Biochemical and histochemical analyses revealed that mutants in both genes have defects in CSPG production. Rescue experiments, gene expression studies, ultrastructural, and mutational analyses all argue that PG mutant chondrocytes undergo precocious maturation, thus inducing early perichondral bone. Our analyses of *in vivo* models for loss of function in *fam20b* or *xylt1* refine previous *in vitro* reports [23], [28] by demonstrating an important role for these PG synthesis genes in cartilage matrix production in intact, developing animals. In closing, we discuss the phenotype of *fam20b* mutant zebrafish with respect to excessive bone formation in Raine syndrome patients, who contain mutations in the paralogous gene *FAM20C* [26], [27].

## Results

### Two mutant loci with increased bone matrix and decreased cartilage matrix phenotypes

**Figure 1:**
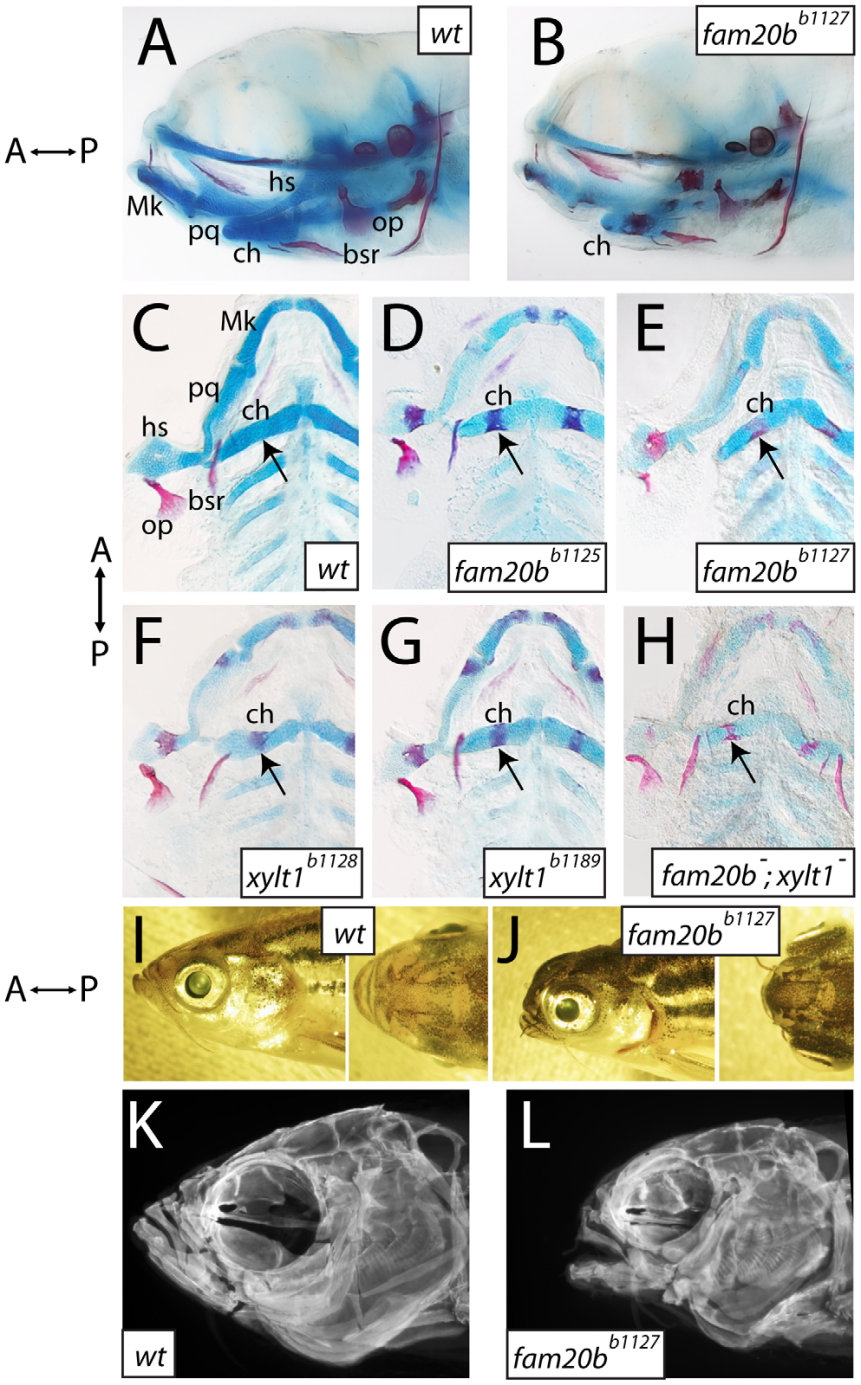
Craniofacial skeletons of mutant zebrafish larvae exhibit increased bone matrix and decreased cartilage matrix. A–H, Alcian blue/Alizarin red-stained 6 dpf larvae, entire heads viewed laterally (A,B, eyes removed) or flat-mounted, dissected pharyngeal skeletons viewed ventrally (C–H). I–L, lateral and dorsal views of entire 8-month-old heads (I,J), and corresponding lateral view images of Alizarin red fluorescence taken by optical projection tomography (OPT; K,L). Overall gross anatomy of the head and craniofacial skeleton are similar in wild types (A) and mutants (B), although staining of cartilage and bone appeared altered in mutants. Compared to wild types (C), *fam20b^b1125^* (D), *fam20b^b1127^* (E), *xylt1^b1128^* (F), and *xylt1^b1189^* (G) mutants had increased Alizarin red staining of bone (arrows) and decreased Alcian blue staining of cartilage. There was no sided-ness to the phenotype, for defective cartilage and bone appeared symmetrically on left and right sides. Double mutant *fam20b^b1127^;xylt1^b1128^* larvae (H) had Alizarin red staining (arrow) similar to that seen in single mutants, but more severe loss of Alcian blue staining. Relative to wild-type siblings (I), *fam20b^b1127^* mutant adults (J) showed foreshortened upper and lower jaws, hypoplastic midface, and bulging eyes. These overall morphological features were underlain by severe reductions to the size of the mutant craniofacial skeleton (K,L). Abbreviations: A = anterior; bsr = branchiostegal ray; ch = ceratohyal; hs = hyosymplectic; Mk = Meckel's; op = opercle; P = posterior; pq = palatoquadrate.

Screening of larval skeletal phenotypes in mutagenized zebrafish revealed a class of mutant with normal gross anatomy at 6 dpf, but with specific defects in the degree of cartilage and bone tissue formation (Figure 1A, 1B). We identified four independently derived lines (*b1125*, *b1127*, *b1128*, and *b1189*), in which homozygous mutants showed increased ossification of many bones, as judged by Alizarin red staining (Figure 1C–1H). In addition, these mutants had decreased Alcian blue staining of cartilage matrix in all cartilaginous elements (Figure 1C–1H), which reflected defects in the production or secretion of cartilage extracellular matrix. Cartilage and bone phenotypes were both present in 100% of mutants examined, although there was variation in the degree to which Alizarin red staining increased. Complementation crosses, scored for these skeletal phenotypes, suggested mutations at two genetic loci among these four mutants. The *b1125* and *b1127* mutations failed a genetic complementation test, producing 51/212 (24%) mutant larvae when heterozygotes were crossed to one another. Likewise, *b1128* and *b1189* failed to complement, producing 60/208 (29%) mutant larvae when heterozygotes were crossed. All other pair-wise complementation crosses produced larvae with wild-type skeletal development. The *b1127;b1128* double mutants showed Alizarin red staining that was similar to each single mutant, although the loss of Alcian blue staining seemed more severe (Figure 1F, quantified below), suggesting that both of these loci drive production of cartilage matrix. Other than these changes to skeletal tissues, overall mutant cranial morphology appeared smaller at 6 dpf (Figure 1A, 1B). In particular, mutant chondral bones appeared shorter than those in wild-type siblings. All homozygous mutants can grow to viable adults, allowing us to investigate whether morphological phenotypes were more apparent at later stages. Compared to wild-type siblings, mutant adults displayed foreshortened upper and lower jaws, hypoplastic midface, and bulging eyes (Figure 1I, 1J). Imaging Alizarin red-stained head skeletons with optical projection tomography (OPT) revealed that morphological defects in adult mutant heads were accompanied by altered craniofacial skeletal morphology (Figure 1K, 1L), predominantly an apparent loss of anterior neurocranial growth.

### Identification of *fam20b* and *xylt1* lesions in skeletal matrix mutants

**Figure 2:**
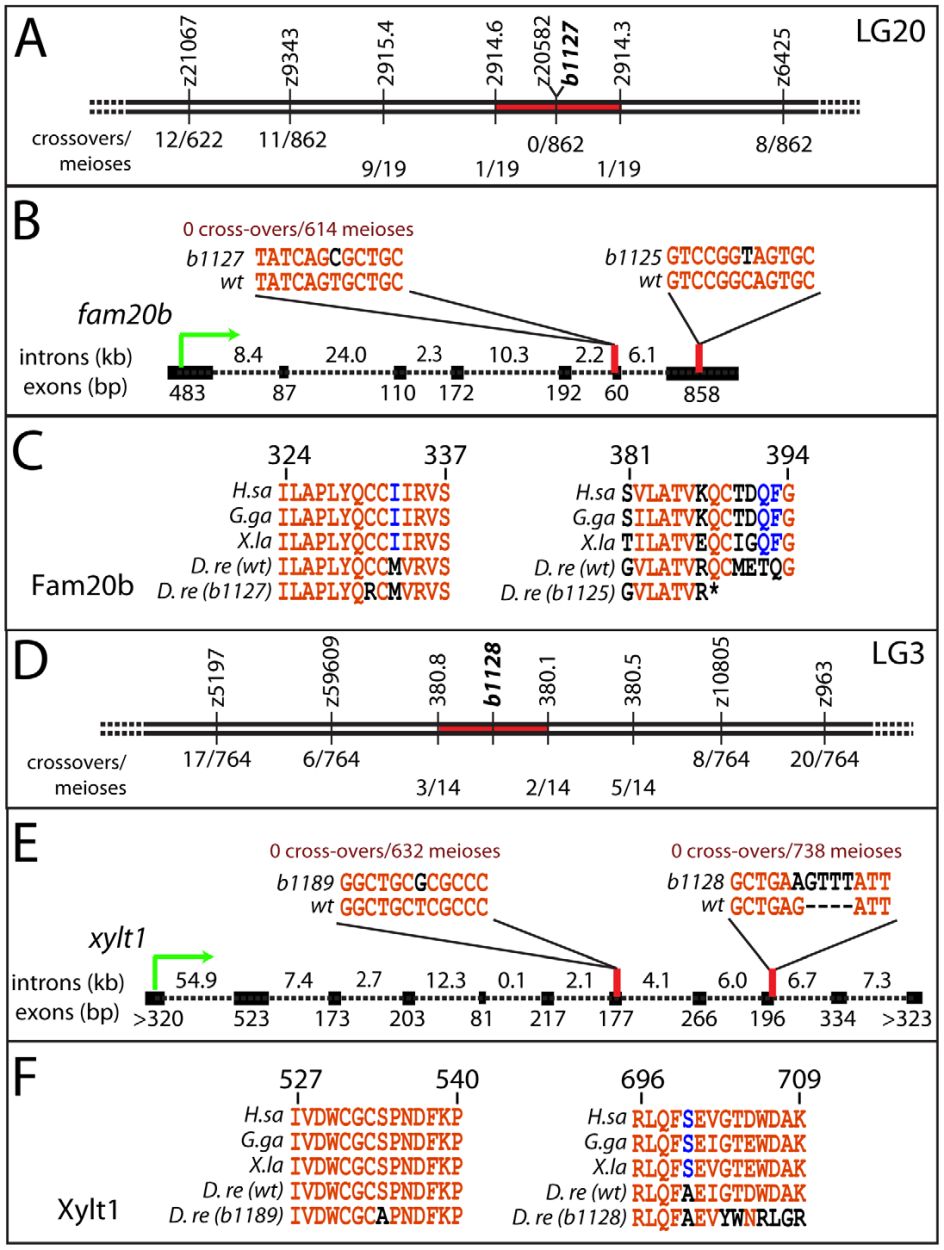
Mapping the mutants reveals lesions in *fam20b* and *xylt1*. A, SSR markers mapped *b1127* to a 0.2 cM interval on LG20 around z20582, showing 0 cross-overs/862 meioses. B, cDNA sequencing of *fam20b^b1127^* revealed a T991C mutation in the sixth exon; genotype assays confirmed perfect linkage of this mutation to mutant phenotype (0 cross-overs/614 meioses). Similarly, *fam20b^b1125^* had a C1162T mutation in the seventh exon. C, The *fam20b^b1127^* mutation changed a highly-conserved Cys to Arg at aa331 (numbering based upon zebrafish protein), while *fam20b^b1125^* mutation changed aa388 from Gln to STOP, which deleted another highly conserved Cys residue at aa389. D, SSR markers mapped *b1128* to a 0.7 cM interval on LG3. E, cDNA sequencing of *xylt1^b1189^* revealed a T1600G mutation in the seventh exon; *xylt1^b1128^* contained a G2103A splice donor mutation in the ninth exon, which forced usage of cryptic splice donors, typically causing a tetranucleotide insertion. Genotype assays confirmed perfect linkage of these mutations to mutant phenotypes (*xylt1^b1189^*: 0 cross-overs/632 meioses; *xylt1^b1128^*: 0 cross-overs/738 meioses). F, *xylt1^b1189^* mutation changed a highly-conserved Ser to Ala at aa534 (numbering based upon zebrafish protein), while *xylt1^b1128^* mutation typically frameshifted the coding sequence from aa702. Lines in A, B, D, and E are not to scale. Abbreviations: *D.re = Danio rerio; G.ga = Gallus gallus; H.sa = Homo sapiens; X.la = Xenopus laevis*.

Genetic mapping confirmed that the two complementation groups of skeletal mutants mapped to independent loci, and sequencing nearby candidates revealed molecular lesions in *fam20b* and *xylt1* that underlie the skeletal phenotype. RAD mapping and subsequent simple sequence repeat (SSR) mapping identified a genetic interval of 0.2 cM on LG20 containing the *b1127* mutation (Figure 2A; [29]; see Materials and Methods). The lesion in *fam20b^b1127^* mutants (991T>C) disrupted a highly conserved cysteine residue (C331R; Figure 2B, 2C; [30]). For the *b1125* allele, cDNA sequencing revealed a 1162C>T mutation in the seventh coding exon of *fam20b^b1125^*, which would alter amino acid 388 from Gln to STOP, thereby truncating the last 22 amino acids, including a highly conserved Cys residue at aa389 (Figure 2B, 2C; [30]). *fam20b^b1125^* transcripts were down-regulated at 55 hours post-fertilization (hpf), while *fam20b^b1127^* transcripts were present at wild-type levels (data not shown), which is consistent with nonsense-mediated RNA degradation [31]. Therefore, mapping and sequence data suggested that mutations within *fam20b* caused the *b1125* and *b1127* phenotypes.

As the first steps in identifying molecular lesions in *xylt1*, RAD mapping and subsequent SSR mapping defined a genetic interval of 0.7 cM on LG3 containing the *b1128* mutation (Figure 2D; [29]). Sequencing of *xylt1* from *b1128* cDNA and gDNA revealed a splice donor mutation (2103G>A) in exon 9 that would produce a frameshifted and truncated C-terminal portion of the zebrafish Xylt1 protein from at least amino acid 702 (out of 919, Figure 2E). For the *b1189* allele, cDNA sequencing revealed a 1600T>G mutation in the seventh coding exon of *xylt1^b1189^*, which altered highly-conserved amino acid 534 from Ser to Ala (Figure 2E, 2F). *xylt1^b1128^* transcripts were down-regulated at 55 hpf, while *xylt1^b1189^* transcripts were present at wild-type levels (data not shown), which is in agreement with nonsense-mediated RNA degradation [31]. These data suggested that the *b1128* and *b1189* phenotypes were caused by mutations in *xylt1*.

### Expressing wild-type *fam20b* rescues *b1125* and *b1127*

**Figure 3:**
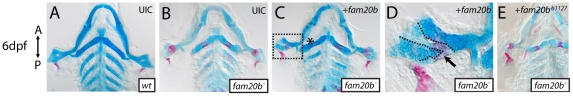
Wild-type *fam20b* expression rescues the *fam20b^b1127^* mutant phenotype. A–E, Flat-mounted, dissected pharyngeal skeletons of Alcian blue/Alizarin red-stained 6 dpf larvae. Both sides of the pharyngeal skeletons are shown because the mosaic nature of the rescue experiment produced asymmetric phenotypes in injected larvae. Compared to uninjected wild-type (A) and mutant (B) controls, *fam20b^b1127^* mutant larvae that were injected with wild-type *fam20b* cDNA under the control of *beta-actin2* promoter (C) showed skeletal elements with rescued cartilage matrix staining by Alcian blue and decreased bone matrix staining (asterisk) by Alizarin red. D, Higher magnification of boxed region in C illustrates patches of light Alcian blue staining surrounded by heavy Alizarin red staining (arrow, dashed lines) adjacent to patches of dark Alcian blue staining surrounded by light Alizarin red staining. E, *fam20b^b1127^* mutants embryos similarly injected with *fam20b^b1127^* did not rescue the mutant skeletal phenotype. Abbreviations: UIC = uninjected control.

We used rescue experiments to test our conclusion that lesions in *fam20b* caused the skeletal phenotypes of *b1125* and *b1127*. Mutant embryos were injected with Tol2 expression plasmids driving expression of wild-type *fam20b*, and then were assayed for skeletal phenotypes. Wild-type *fam20b* cDNA rescued *fam20b^b1127^* (n = 10/13) and *fam20b^b1125^* (n = 3/8) skeletal phenotypes when driven by the ubiquitous *beta-actin2* promoter (Figure 3A–3D, data not shown; [32]). There was variation in the degree to which the entire skeleton, or even an entire skeletal element, was rescued, which would be expected from mosaicism inherent to the transient injection protocol. We observed in these rescues a correlation between chondrocytes surrounded by faint Alcian blue staining and overlying bone (n = 11 skeletal elements). That is, in adjacent patches of chondrocytes, lighter stained Alcian blue matrix was covered with abundant Alizarin red staining, whereas darker stained Alcian blue matrix was not surrounded by heavy Alizarin red staining (Figure 3D, dashed lines), a finding that will be of significance below. No differences in skeletal phenotypes at 6 dpf were observed in control wild-type embryos that were injected with wild-type *fam20b* expression constructs (Figure S1A). Also, the mutant skeletal phenotype was not rescued when *fam20b^b1127^* mutant embryos were injected with Tol2 expression plasmids containing mutant *fam20b^b1127^* (n = 0/23) or *fam20b^b1125^* (n = 0/12) cDNA (Figure 3E, data not shown). To address whether over-expression of *fam20b* was having non-specific effects, we subjected *xylt1* mutants to Tol2-mediated *fam20b* over-expression. Wild-type *fam20b* was not sufficient to rescue skeletal defects in *xylt1^b1128^* mutants (n = 0/11; Figure S1B). Experiments injecting *xylt1^b1128^* mutants with wild-type *xylt1* expression constructs did not lead to rescue of the skeletal phenotype (data not shown), which may be due to the fact that our *xylt1* cDNA constructs contained a transmembrane domain that was removed from similar mis-expression studies [28], [33].

As measured by Alcian blue staining, PG secretion by developing chondrocytes in the anterior pharyngeal arches was not detected at 48 hours post-fertilization (hpf), but was abundant at 60 hpf (Figure S2). To understand when *fam20b* plays a role during development, wild-type *fam20b* expression was induced under the control of a heat-shock-inducible promoter (*hsp70l*; [32]). A single induction of wild-type *fam20b* at 55 hpf proved sufficient to rescue skeletal phenotypes of *fam20b^b1125^* mutant larvae at 6 dpf (n = 6/7; Figure S1C–S1E), although again there was variation in the ability of a transient injection to rescue completely even a given skeletal element. In summary, we conclude that *fam20b* is the mutated gene in *b1125* and *b1127*, and our data argue that the timing of *fam20b* action in producing the craniofacial skeleton correlates with the onset of overt chondrogenesis.

### *fam20b* and *xylt1* are expressed in developing chondrocytes

**Figure 4:**
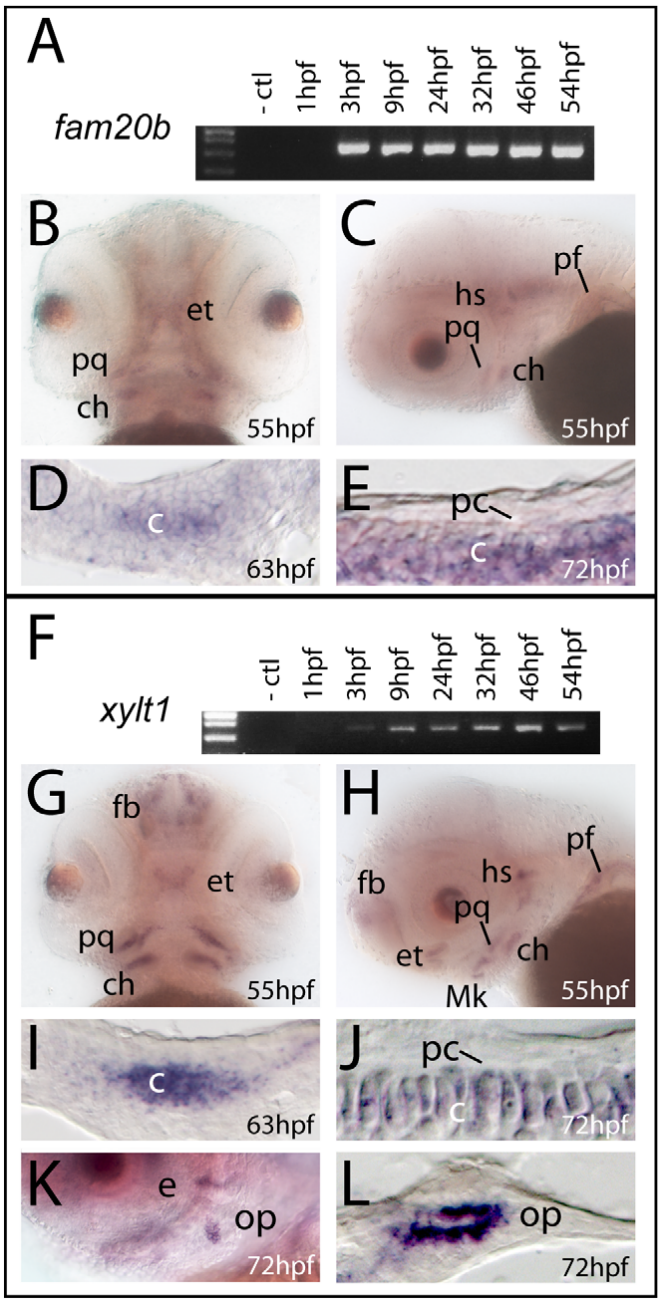
*fam20b* and *xylt1* are expressed in chondrocytes, but not in perichondrium. A,F, RT-PCR on wild-type extracts at ages indicated; B,C,G,H,K, whole-mount and D,E,I,J,L section *in situ* hybridization on wild-type embryos. RT-PCR demonstrated transcripts for *fam20b* (A) and *xylt1* (F) from 3 hpf through 54 hpf. Frontal and lateral views of embryos stained by whole-mount *in situ* hybridization revealed transcripts for *fam20b* (B,C) and *xylt1* (G,H) in developing cartilage elements of the craniofacial and pectoral fin skeletons at 55 hpf. In addition, there was diffuse expression of *fam20b* in the brain and specific *xylt1* expression in the forebrain (fb). Horizontal section *in situ* hybridization localized transcripts for *fam20b* and *xylt1* to developing chondrocytes (c) of the ceratohyal at 63 hpf (D,I) and 72 hpf (E,J), but no expression in perichondrium (pc) was detected. Lateral view of whole-mount (K) and horizontal section (L) *in situ* hybridization revealed expression of *xylt1* in osteoblasts of the opercle (op) at 72 hpf. Abbreviations: -ctl = negative control; c = chondrocytes; ch = ceratohyal; e = eye; et = ethmoid; fb = forebrain; hpf = hours post-fertilization; hs = hyosymplectic; Mk = Meckel's; op = opercle; pc = perichondrium; pf = pectoral fin; pq = palatoquadrate.

Transcripts for *fam20b* and *xylt1* in wild-type larvae were detected by RT-PCR from 3 hpf through 54 hpf (Figure 4A, 4F), demonstrating that *fam20b* and *xylt1* were expressed during the mid-blastula transition and early skeletogenic time points. *In situ* hybridization revealed *fam20b* and *xylt1* expression in chondrocytes of developing skeletal elements at 53 hpf, 63 hpf, and 72 hpf, but these levels decreased by 4 dpf (Figure 4B–4E, 4G–4J, data not shown). *xylt1* transcripts were readily apparent in osteoblasts of newly-forming dermal bone at 3 dpf (Figure 4K, 4L), which is interesting to consider under the proposition that dermal bones progress through a transient chondrogenic phase [34]. However, neither *fam20b* nor *xylt1* transcripts were detected in developing perichondrium at 3 dpf, 4 dpf, or 6 dpf, which spanned the time perichondral osteoblasts appear (Figure 4D, 4E, 4I, 4J, and data not shown). Apart from their up-regulated expression in skeletal tissues, *fam20b* showed diffuse ubiquitous expression in brain and craniofacial mesenchyme, and *xylt1* transcripts were expressed in discrete domains of the brain, such as the developing forebrain (Figure 4B, 4C, 4G, 4H). In summary, *fam20b* and *xylt1* are expressed in developing chondrocytes, but not in cells of the perichondrium, even at stages when we know bone-forming cells have differentiated within this tissue. Our findings are thus consistent with the notion that mutations in these genes act directly in chondrocytes to produce the mutant cartilage phenotype, but only indirectly in causing elevated perichondral bone.

### *fam20b* and *xylt1* mutants exhibit partial loss of cartilage PGs

**Figure 5:**
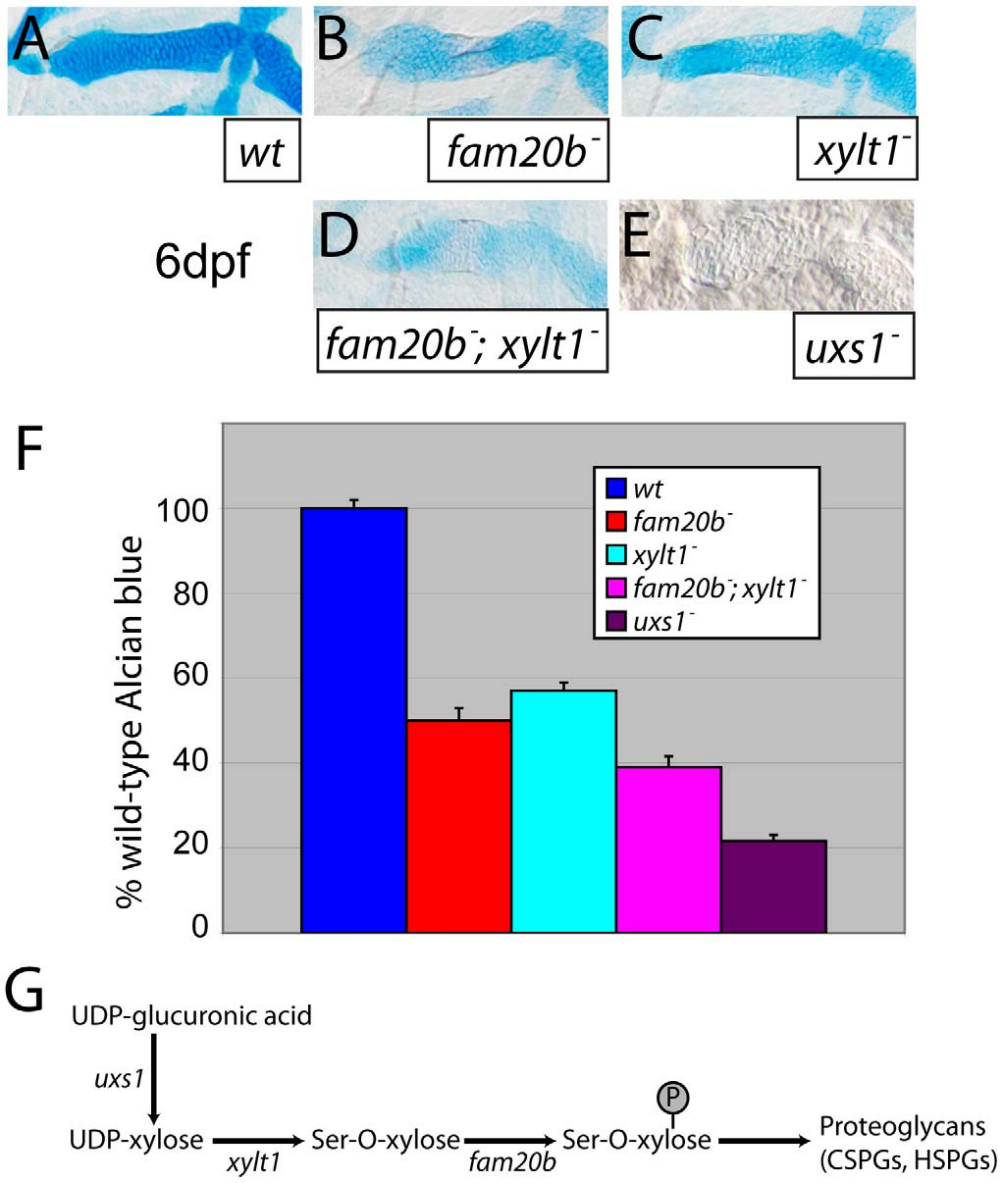
Reduction of Alcian blue staining in *fam20b* and *xylt1* mutants is less severe than in *uxs1* mutants. A–E, Alcian blue-stained 6 dpf ceratohyals. Compared to wild types (A), Alcian blue staining appeared lower in *fam20b^b1127^* (B) and *xylt1^b1128^* (C) mutants. Staining in *fam20b^b1127^;xylt1^b1128^* double mutants (D) was decreased in comparison to single mutants, although *uxs1* mutants (E) showed the greatest decrease in Alcian blue staining. F, Quantitation of Alcian blue in lysates of 6 dpf larvae revealed statistically significant changes (ANOVA p<0.0001) for each mutant class. Compared to wild-type Alcian blue staining, *fam20b^b1127^* mutants had 50±3.0%, *xylt1^b1128^* mutants had 57±2.0%, *fam20b^b1127^;xylt1^b1128^* double mutants had 39±2.6%, and *uxs1* mutants had 22±1.5%. G, Simplified pathway of PG synthesis, demonstrating that *uxs1* is upstream of both *xylt1*, which adds xylose to a serine (Ser) residue of the core protein, and also *fam20b*, which phosphorylates (P) xylose in the nascent GAG chain. Abbreviations: CSPGs = chondroitin sulfate proteoglycans; HSPGs = heparan sulfate proteoglycans.

Quantifying spectrometrically lysates from at least three Alcian blue-stained clutches for *fam20b* or *xylt1* single mutants, or *fam20b;xylt1* double mutants (see Materials and Methods; [35]), we found statistically significant changes in Alcian blue levels between all genotypes tested (Figure 5A–5D, 5F; ANOVA p<0.0001). Levels of Alcian blue in *fam20b* and *xylt1* mutants were 50±3.0% and 57±2.0% of their wild-type siblings, respectively, while *fam20b;xylt1* double mutants further reduced Alcian blue levels to 39±2.6% of their wild-type siblings (Figure 5F). Recently, we showed that a null mutation in *UDP-xylose synthase1* (*uxs1*) abolished zygotic production of UDP-xylose [18], a sugar that ultimately is the substrate for both Fam20b and Xylt1 during PG synthesis (Figure 5G; [22], [23]). We used the *uxs1* mutant to estimate whether *fam20b* and *xylt1* mutants were null alleles. Relative to *fam20b* and *xylt1* single and double mutants, cartilages from *uxs1* mutants showed even less Alcian blue staining (Figure 5E), reducing levels to 22±1.5% of those in wild-type siblings (Figure 5F). Therefore, quantitiative comparisons with *uxs1* mutants suggested that the *fam20b^b1127^* and *xylt1^b1128^* alleles did not completely eliminate cartilage PG production; even *fam20b;xylt1* double mutants were less severe than *uxs1* mutants.

**Figure 6:**
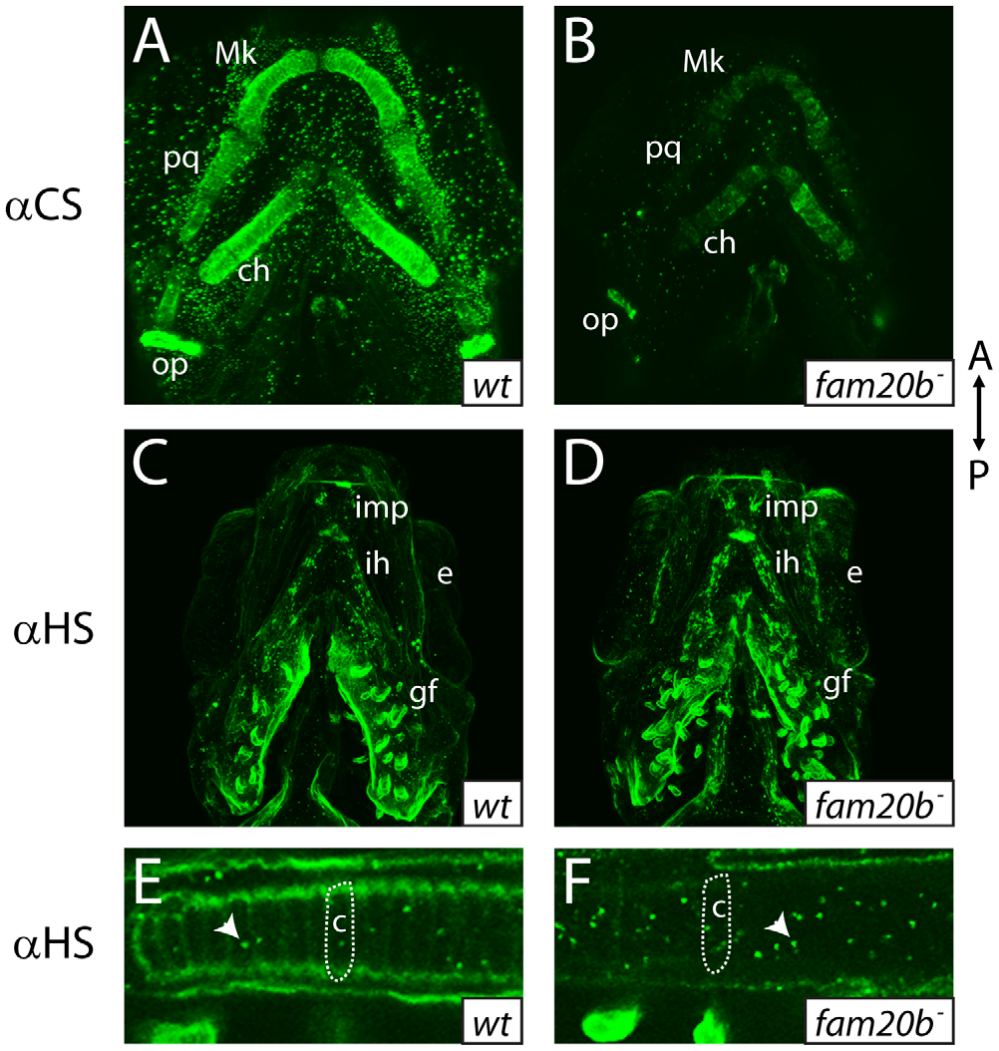
*fam20b* mutants display reduced proteoglycan immunoreactivity A–F, Ventral views of confocal images of embryonic heads immunostained for chondroitin sulfate proteoglycan (CSPG) at 3 dpf (A,B) or heparan sulfate PG (HSPG) at 4 dpf (C–F). Compared to abundant CSPGs detected in wild-type skeletal elements (A), *fam20b^b1127^* mutants showed decreased CSPG immunoreactivity (B). HSPGs were abundant in gill filaments and ventral muscle groups of both wild-type (C) and *fam20b^b1127^* mutant zebrafish (D). HSPG immunoreactivity was reduced greatly around developing chondrocytes of the *fam20b^b1127^* mutant ceratohyal (outline in F), compared to those of wild-type siblings (outline in E). Also, the number of intracellular HSPG aggregates (arrowhead) was increased in *fam20b^b1127^* mutant chondrocytes. Abbreviations: A = anterior; αCS = anti-chondroitin sulfate; αHS = anti-heparan sulfate; c = chondrocyte; ch = ceratohyal; e = eye; gf = gill filament; ih = interhyoideus; imp = intermandibularis posterior; Mk = Meckel's; op = opercle; P = posterior; pq = palatoquadrate.

To show by an independent method that *fam20b* and *xylt1* mutants had cartilage PG defects that were less severe than *uxs1* mutants, we analyzed GAG disaccharide levels using biochemical and immunohistochemical methods. Both heparan sulfate PGs (HSPGs) and chondroitin sulfate PGs (CSPGs) are xylose-dependent, so both of these classes of PG may be affected in *fam20b* and *xylt1* mutants. HPLC analyses on lysates of whole 5 dpf larvae demonstrated that *fam20b* and *xylt1* mutants had decreased levels of CSPG disaccharides, which are predominant in cartilage [36], and again, the losses were less severe than seen in *uxs1* mutants (Figure S3). HSPG disaccharide levels were not affected consistently among these mutants; *fam20b* and *uxs1* mutants showed decreases, whereas *xylt1* mutants did not (Figure S3). Consistent with the biochemical data, immunodetection assays revealed losses of both CSPGs and HSPGs specifically around developing chondrocytes in *fam20b* mutants (Figure 6A–6F). No loss of CSPGs in *xylt1* mutant larvae were detected (data not shown), suggesting that the CSPG antibody may recognize epitopes still present in *xylt1* mutants. Together, these biochemical and immunohistochemical data revealed cartilage PG defects in *fam20b* and *xylt1* mutants. Furthermore, the quantitative comparisons with *uxs1* mutant embryos suggested either that *fam20b^b1127^* and *xylt1^b1128^* are not null alleles, or that redundant genes compensate for their loss of function (see Discussion).

**Figure 7:**
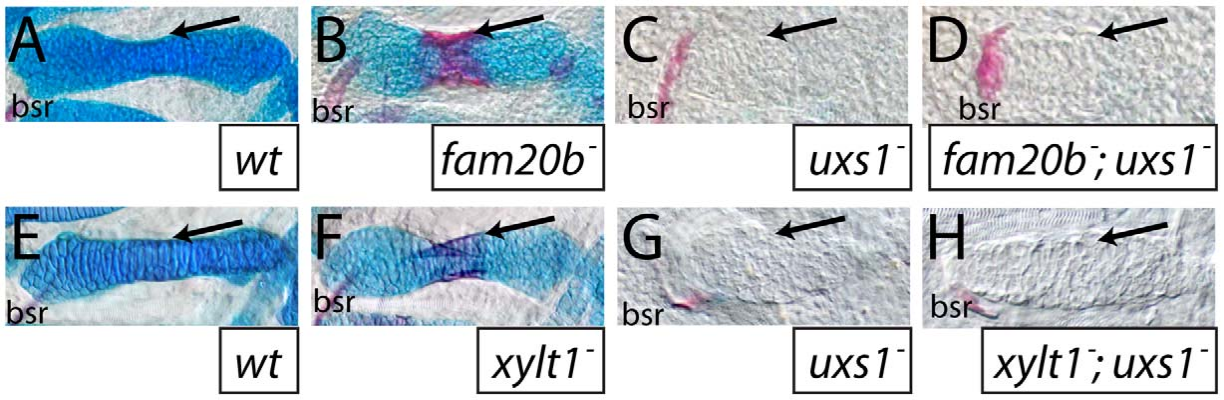
The *uxs1* mutation is epistatic to cartilage and bone phenotypes of *fam20b* and *xylt1* mutants. A–H, Alcian blue/Alizarin red-stained 6 dpf ceratohyals. Compared to wild types (A,E), cartilage matrix of *fam20b^b1127^* (B) and *xylt1^b1128^* (F) mutants stained less with Alcian blue, but the perichondria of *fam20b* and *xylt1* mutants stained more with Alizarin red (arrows) than in wild types. Similar to *uxs1* mutants (C,G), *uxs1;fam20b^b1127^* (D) and *uxs1;xylt1^b1128^* (H) double mutants showed a greater decrease in Alcian blue staining of cartilage matrix than the decrease seen in *fam20b* and *xylt1* mutants; also, Alizarin red staining (arrows) in *uxs1* single mutant and *uxs1;fam20b* and *uxs1;xylt1* double mutant perichondria was at wild-type levels. Abbreviations: bsr = branchiostegal ray.

Since both Fam20b and Xylt1 depend upon UDP-xylose, and hence *uxs1* function, in order to promote GAG synthesis (Figure 5G), loss of *uxs1* function should mask the skeletal phenotypes of *fam20b* and *xylt1* embryos. Consistent with *fam20b* and *xylt1* being downstream of *uxs1*, skeletal phenotypes of *fam20b;uxs1* and *xylt1;uxs1* larvae appeared identical to *uxs1* single mutants, including severe loss of Alcian blue staining and undetectable levels of perichondral bone, as predicted (Figure 7A–7H). Despite the strong *uxs1* mutant phenotype, elimination of just one copy of *uxs1* did not sensitize zebrafish skeletons to the loss of *fam20b* or *xylt1*. Trans-heterozygous larvae displayed normal skeletal phenotypes, and no enhancement of the homozygous *fam20b* and *xylt1* mutant phenotypes was observed when larvae also were heterozygous for the *uxs1* mutation (data not shown). These epistasis experiments provided genetic support that *fam20b* and *xylt1* function in the PG synthesis pathway *in vivo*.

### Mutants have increased bone due to precocious differentiation of perichondral osteoblasts

**Figure 8:**
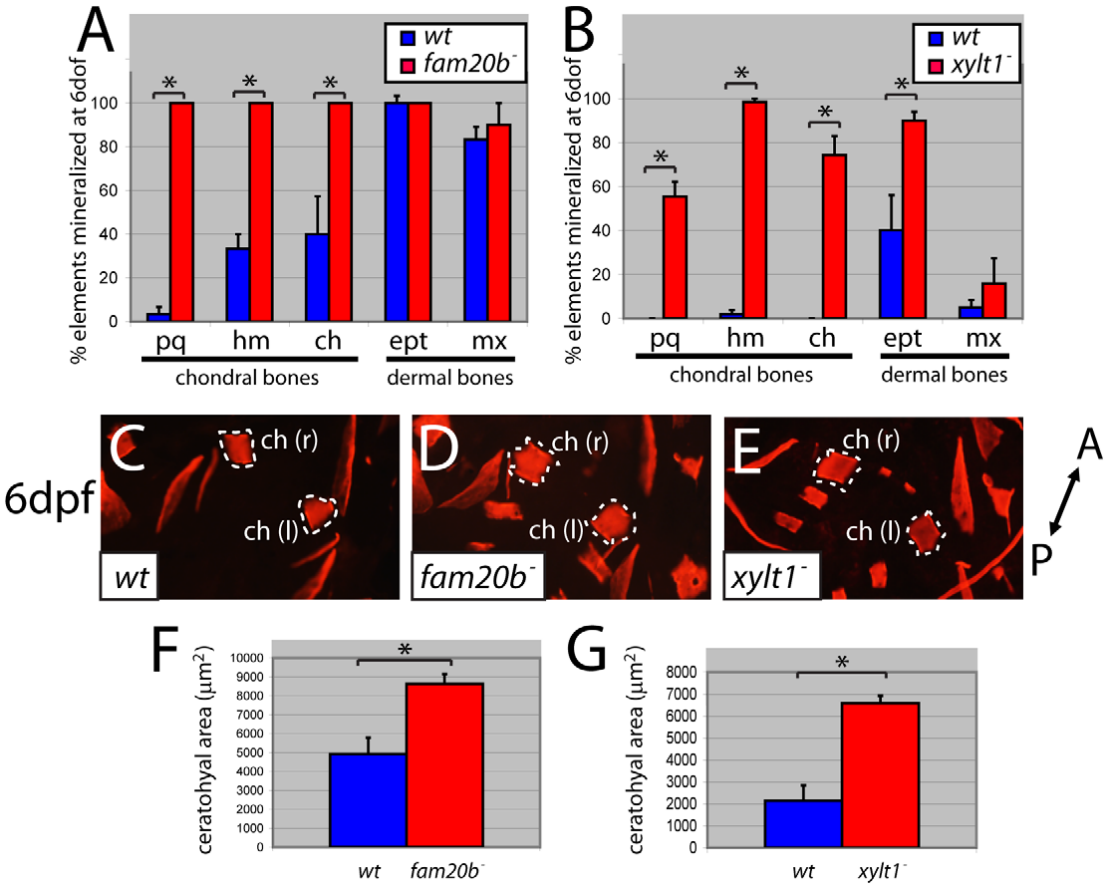
*fam20b* and *xylt1* mutants have increased perichondral bone. At 6 dpf, Alizarin red staining (see Figure 1C–1G) was visible more often in skeletal elements of *fam20b^b1127^* (A) and *xylt1^b1128^* (B) mutants than in wild types. Statistically significant (*, p<0.05) increases, however, were observed in more chondral bones (pq, hm, ch) than dermal bones (ept, mx). Quantitative analyses of the sum of bone areas in left and right ceratohyals (ch(l) and ch(r), dashed outlines) in whole-mount, ventral images of live Alizarin red fluorescence (C–E) revealed statistically significant (*, p<0.05) increases in *fam20b^b1127^* (F) and *xylt1^b1128^* (G) mutants, compared to wild-type siblings at 6 dpf. Abbreviations: A = anterior; ch = ceratohyal; ept = entopterygoid; hm = hyomandibular; l = left; mx = maxilla; pq = palatoquadrate; P = posterior; r = right.

**Figure 9:**
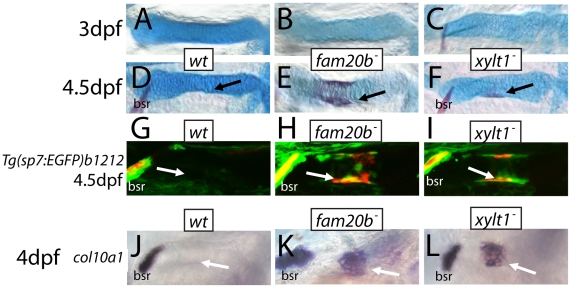
Precocious bone formation and osteoblast differentiation in perichondria of *fam20b* and *xylt1* mutants. A–F, Alcian blue/Alizarin red-stained ceratohyals. G–I, live Alizarin red fluorescence of *fam20b^b1127^;Tg(sp7:EGFP)b1212* and *xylt1^b1128^;Tg(sp7:EGFP)b1212* larvae. J–O, whole-mount *in situ* hybridization of 4 dpf ceratohyals. No signs of Alizarin red staining were observed at 3 dpf in ceratohyals of wild types (A), or *fam20b^b1127^* (B) or *xylt1^b1128^* (C) mutants. By 4.5 dpf, Alizarin red staining was still absent from ceratohyals of wild-type larvae (D, arrow), although it was detected in ceratohyals of *fam20b^b1127^* (E, arrow) and *xylt1^b1128^* (F, arrow) mutants. Both GFP expression and Alizarin red staining were at background levels in the perichondrium of the ceratohyal in 4.5 dpf wild types (G, arrow), but were obvious in the *fam20b^b1127^* and *xylt1^b1128^* mutant perichondria (H,I, arrow). At 4 dpf, *col10a1* expression was not detected in the ceratohyal perichondrium of wild types (J, arrow), but was expressed highly in *fam20b^b1127^* (K, arrow) and *xylt1^b1128^* (L, arrow) mutant perichondria. Abbreviations: bsr = branchiostegal ray.

We find that two parameters of bone matrix production along the endochondral ossification pathway are increased in both *fam20b* and *xylt1* mutants, which is in contrast to decreased production of cartilage matrix. First, the frequency of chondral bone Alizarin red staining at 6 dpf was increased (see Figure 1C–1H for example of material scored), whereas dermal bone staining did not consistently show statistically significant effects (Figure 8A, 8B). Second, the amount of staining in a given chondral bone (e.g., the ceratohyal) was also increased significantly in *fam20b* and *xylt1* mutants (Figure 8C–8G). The increased bone resided on the inner surface of the perichondrium (see Figure 9H, 9I), as expected for chondral, rather than dermal, bones. Therefore, *fam20b* and *xylt1* mutants showed specific increases in the amount of perichondral bone.

The observed increase in perichondral bone might result from three possible scenarios: 1) osteoblasts differentiate at the correct time, but exhibit enhanced secretory activity; 2) osteoblasts differentiate at the correct time, but a larger pool of pre-osteoblasts exists in mutant perichondria; or 3) osteoblasts differentiate early. Examination of skeletal phenotypes and cellular and molecular markers of osteoblasts at time points prior to 6 dpf demonstrated that the increase in mutant perichondral bone resulted from early initiation of osteogenesis. While neither mutants nor wild types showed signs of perichondral bone at 3 dpf (Figure 9A–9C), Alizarin red staining was observed in chondral bones of *fam20b* and *xylt1* mutants, but not wild types, at 4.5 dpf (Figure 9D–9F, arrows). Bone formation was not accelerated in time further in *fam20b;xylt1* double mutants, as perichondral bone was not detected at 3 dpf, but was prominent by 4.5 dpf (data not shown). To address the cellular basis for early perichondral bone, *fam20b* and *xylt1* mutants were bred into transgenic zebrafish expressing EGFP under a promoter that is restricted to osteoblasts (*Tg(sp7:EGFP)b1212*; [37]). Transgenic mutants showed GFP expression and Alizarin red fluorescence in the perichondrium of chondral bones by 4.5 dpf, whereas their wild-type siblings showed neither of these markers at this time point (Figure 9G, 9H, arrows). Further support for the temporal shift in bone development came from similar analyses at 6 dpf, when wild types demonstrated levels of GFP and Alizarin red staining that were comparable to those observed in the perichondrium of mutant chondral bones at 4.5 dpf (data not shown). Expression of molecular markers of osteoblasts, such as *col10a1* and *runx2b*, was increased in the perichondrium of mutant chondral bones at 4 dpf, compared to that seen in their wild-type siblings (Figure 9J–9L, arrows, data not shown). In total, these data argue that the cellular basis of excessive bone in *fam20b* and *xylt1* mutants is the precocious differentiation of secretory osteoblasts in mutant perichondria.

### Early chondrocyte maturation promotes precocious perichondral bone in PG mutants

**Figure 10:**
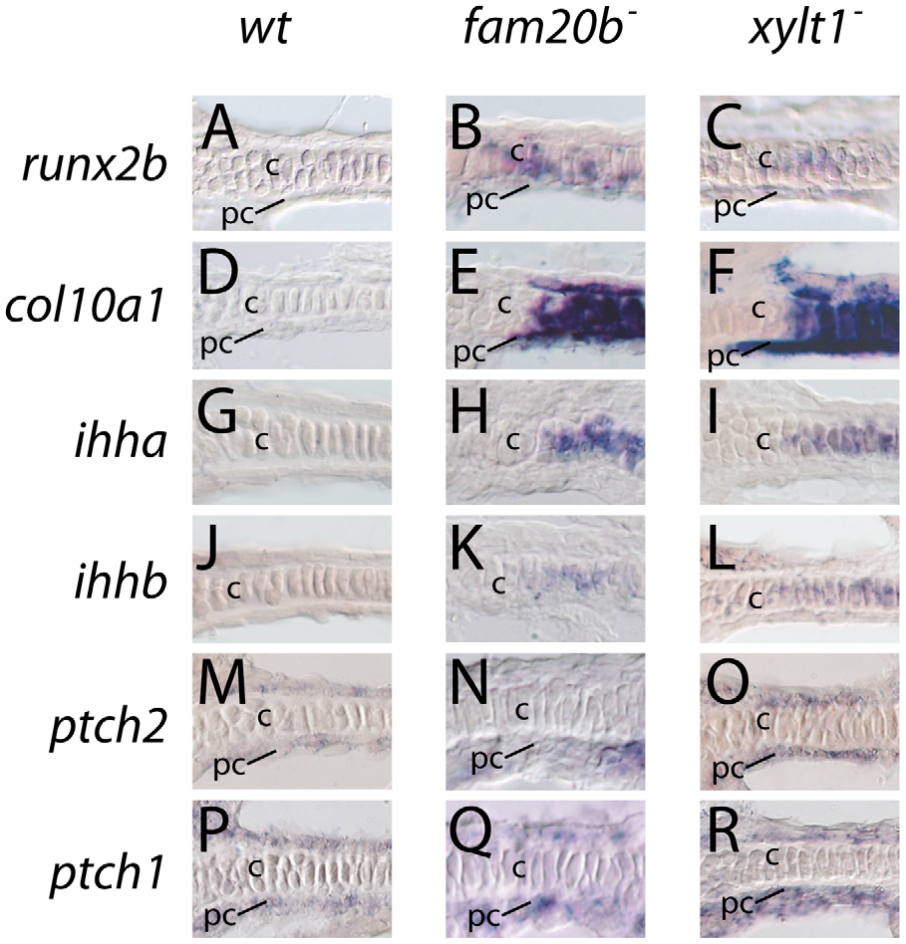
Early molecular markers of chondrocyte maturation in *fam20b* and *xylt1* mutants. A–R, horizontal section *in situ* hybridization of developing ceratohyals. The levels of *runx2b* transcripts appeared higher in chondrocytes (c) and perichondrium (pc) of the ceratohyal in *fam20b^b1127^* (B) and *xylt1^b1128^* (C) mutants, than in wild types (A) at 72 hpf. Expression of *col10a1* was abundant in chondrocytes (c) and perichondrium (pc) of the ceratohyal in *fam20b^b1127^* (E) and *xylt1^b1128^* (F) mutants, but was not detectable in wild types (D) at 83 hpf. Levels of *ihha* and *ihhb* transcripts were up-regulated in chondrocytes (c) of the ceratohyal in *fam20b^b1127^* (H,K) and *xylt1^b1128^* (I,L) mutants, but were not detectable in wild types (G,J) at 72 hpf. Transcript levels for *ptch2* were increased in the perichondrium of *fam20b^b1127^* (N) and *xylt1^b1128^* (O) mutants, compared to wild types (M) at 72 hpf, whereas no obvious differences in levels of *ptch1* expression in perichondria were apparent (P–R). Abbreviations: c = chondrocytes; pc = perichondrium.

Our expression data (Figure 4C, 4F), biochemical data (Figure 5F, Figure S3), and rescue experiments (Figure 3C, 3D) suggest that the premature perichondral bone in *fam20b* and *xylt1* mutants is caused by chondrocyte defects. Indian hedgehog (Ihh) is an osteo-inductive signal expressed by chondrocytes as they mature, and is required for perichondral bone formation [7], [8], [38]. Therefore, we hypothesized that Ihh and other markers of chondrocyte maturation would appear earlier in *fam20b* and *xylt1* mutants. The transcription factor Runx2 positively regulates chondrocyte maturation through transcriptional activation of such genes as *col10a1* and *ihh* [39]–[41], so our hypothesis predicted premature up-regulation of *runx2* genes and their downstream targets. In support, transcript levels for *runx2b* were up-regulated in mutant chondrocytes at 3 dpf compared to wild-type chondrocytes (Figure 10A–10C), and *col10a1*, *ihha*, and *ihhb* expression was up-regulated in mutant chondrocytes earlier than in chondrocytes of wild-type siblings (Figure 10D–10L). Expression of *sox9a* and *col2a1a* was high in both *xylt1* mutant and wild-type chondrocytes at 3 dpf (data not shown). On the other hand, both transcripts were down-regulated in mature chondrocytes by 6 dpf (data not shown), suggesting that some markers of chondrocyte gene expression were not affected in PG mutants. Importantly, the initiation of chondrocyte differentiation was not accelerated overall in mutants, as Alcian blue staining of pharyngeal cartilages was absent at 48 hpf and present at 60 hpf both in wild types and in PG mutants (Figure S2). Therefore, the timing of chondrocyte differentiation was accelerated only after mutant cells had begun to immerse themselves in PG-rich extracellular matrix. The relative timing of these events is consistent with our ability to rescue the *fam20b* mutant phenotype with induction of wild-type *fam20b* at 55 hpf (see Figure S1).

**Figure 11:**
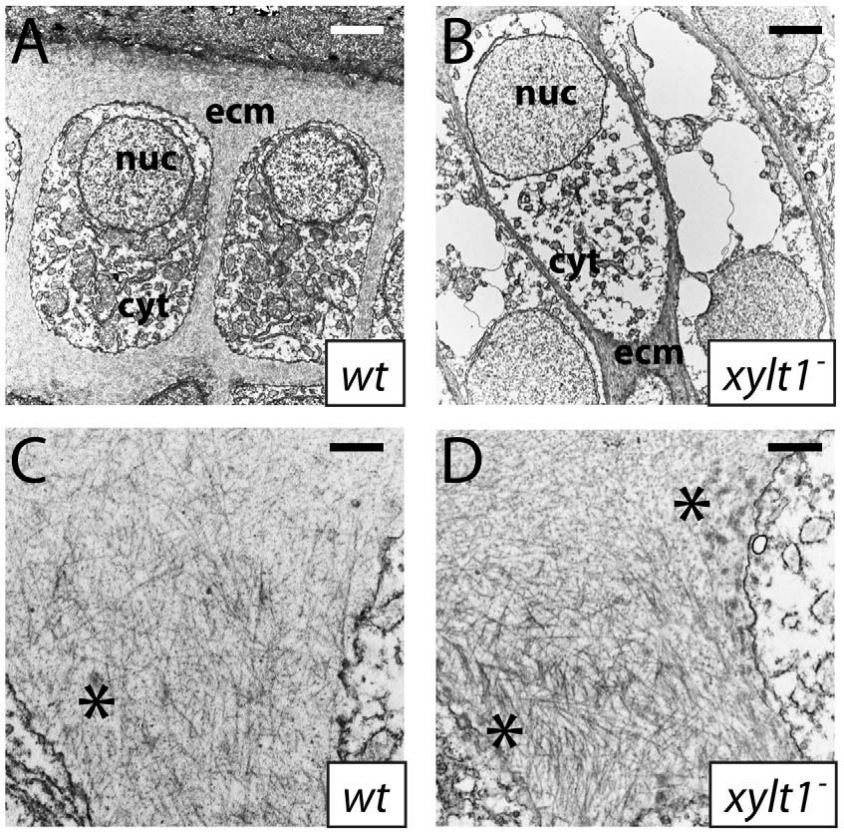
Ultrastructural evidence of premature chondrocyte hypertrophy and aberrant matrix production in *xylt1* mutants. A–D, transmission electron micrographs of 84 hpf ceratohyals. At 5600X magnification, chondrocytes in the central region of the wild-type ceratohyal displayed abundant rough ER, Golgi, and mitochondria in the cytoplasm (A). Similar views of *xylt1* mutant chondrocytes at the same age and comparable positions within the developing ceratohyal showed cytoplasmic clearing and tremendous reduction in biosynthetic organelles (B). Mutant chondrocytes also appeared to be separated by less extracellular matrix. While wild-type (C) and mutant (D) extracellular matrix demonstrated fibrillar collagens at 31000X magnification, mutant matrix showed tighter fibril packing and also contained more amorphous electron-dense structures (*) in pericellular matrix than seen in wild types. Abbreviations: cyt = cytoplasm; ecm = extracellular matrix; nuc = nucleus. Scale bars: A,B = 2 µm; C,D = 500 nm.

Given that PG mutant chondrocytes expressed molecular markers of maturation prematurely, we performed ultrastructural analyses to assay whether PG mutant chondrocytes showed early cellular features of chondrocyte hypertrophy. Examined by transmission electron microscopy at 84 hpf, wild-type chondrocytes in the central portion of the ceratohyal contained abundant rough ER, Golgi complexes, and mitochondria, indicative of their high biosynthetic and secretory activity (Figure 11A; [42]). By comparison, *xylt1* mutant chondrocytes in the central portion of the ceratohyal at 84 hpf displayed ultrastructural hallmarks of hypertrophic differentiation, including increased cell size, reduction in number of biosynthetic organelles, and cytoplasmic clearing (Figure 11B). Although chondrocytes along almost the entire ceratohyal showed premature hypertrophy in mutants, the most pronounced changes in cellular morphology were in the center of this cartilage element, consistent with the timing and location of molecular changes in gene expression (see Figure 10). Near the end of the *xytl1* mutant ceratohyal, we observed chondrocytes that appeared like those in the center of the wild-type ceratohyal (data not shown). Furthermore, we observed differences between wild type and mutants in cartilage ECM ultrastructure. Wild-type cartilage ECM contained well-dispersed matrix proteins within fibrillar collagen, forming a well-defined ECM network (Figure 11C). Both territorial (pericellular) and interterritorial (outer) matrix showed uniform organization. In contrast, *xylt1* mutant cartilage ECM was electron dense, tightly packed, and highly fibrillar (Figure 11D). As a result, *xylt1* mutant chondrocytes were closer to each other than the cells in WT cartilage (Figure 11B). Furthermore, territorial matrix lateral to *xylt1* mutant chondrocytes contained more poorly defined, electron dense precipitates 20–50 nm in diameter than seen in wild-type matrix (* in Figure 11C, 11D). In addition to verifying matrix defects, our ultrastructural analyses demonstrated that *xylt1* mutant chondrocytes displayed cellular hallmarks of hypertrophic differentiation prior to those of wild-type siblings.

**Figure 12:**
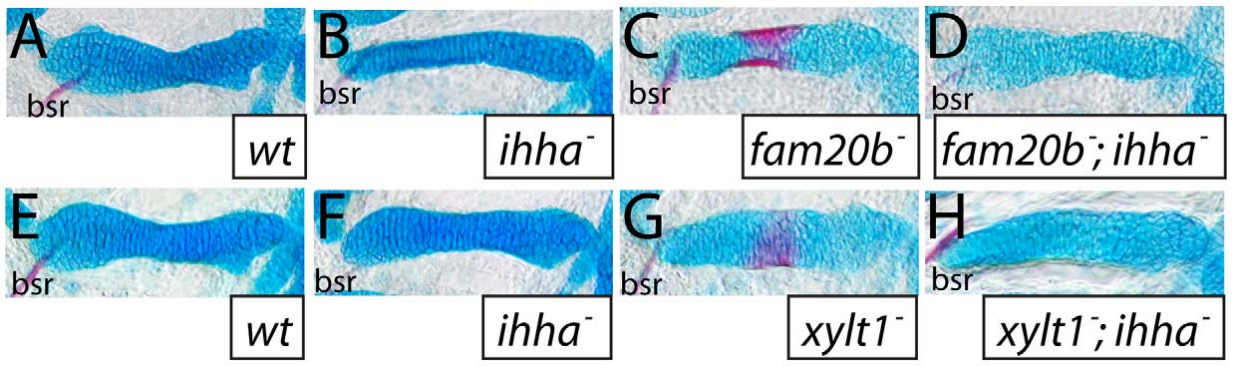
The *ihha* mutation is epistatic to bone, but not cartilage, phenotypes of *fam20b* and *xylt1* mutants. A–H, Alcian blue/Alizarin red-stained 6 dpf ceratohyals. Alcian blue staining was comparable between wild-type (A,E) and *ihha* mutant (B,F) cartilages, but was decreased in *fam20b^b1127^* (C) and *xylt1^b1128^* (G) single mutant, and in *fam20b^b1127^;ihha* (D) and *xylt1^b1128^;ihha* (H) double mutant, cartilages. Alizarin red staining of *fam20b^b1127^* (C) and *xylt1^b1128^* (D) chondral bones was abundant, while no such staining was observed in wild types (A,E), *ihha* mutants (B,F), or *fam20b^b1127^;ihha* (D) and *xylt1^b1128^;ihha* double mutants (H). Abbreviations: bsr = branchiostegal ray.

Given these accelerated molecular and cellular features of chondrocyte maturation in PG mutants, we used molecular and genetic means to demonstrate that chondrocyte Ihh expression signaled prematurely to induce perichondral bone. Expression of *ptch2*, a downstream marker of Hh signaling, was increased in ceratohyal perichondrium of *fam20b* and *xylt1* mutants at 3 dpf (Figure 10M–10O), suggesting increased Ihh signaling in PG mutants. No differences in perichondral *ptch1*, *gli2*, and *gli3* expression were observed between *xylt1* or *fam20b* mutants and wild types (Figure 10P–10R, data not shown), perhaps reflecting the notion that potential transcriptional targets of Hh signaling are not employed in every cell type [43]. Because *ihha* mutant zebrafish have delayed perichondral ossification [7], we could test the functional significance of early *ihha* expression by creating *fam20b;ihha* and *xylt1;ihha* double mutants. Perichondral bone was suppressed in *fam20b;ihha* and *xylt1;ihha* double mutants (Figure 12A–12H), showing that *ihha* is required for early perichondral bone in *fam20b* and *xylt1* mutants. While epistatic to the bone phenotypes, the *ihha* mutation did not alter cartilage matrix reduction of *fam20b* and *xylt1* mutants, consistent with the interpretation that *ihha* acts downstream of the cartilage matrix defects. In summary, these data argue that cartilage PG defects in *fam20b* and *xylt1* mutants primarily accelerated the timing of chondrocyte maturation and *ihha* expression, which then secondarily triggered early perichondral bone formation.

## Discussion

Investigation of the vertebrate skeletal system has revealed substantial insight into the structural and functional roles of proteoglycans (PGs; [14]–[16], [18]–[20]). Here, we provide experimental data linking functions of long-studied (Xylt1) and recently-identified (Fam20b) members of the PG synthesis pathway to skeletal development *in vivo*. Xylosyltransferases (Xylts) have been known for 40 years to initiate glycosaminoglycan side chain outgrowth onto protein cores of PGs by transferring xylose to serine residues (Figure 5G; [22], [44]). Based on tissue culture and biochemical assays, Xylts were shown to function in the synthesis of both heparan sulfate PGs (HSPGs) and chondroitin sulfate PGs (CSPGs; [28], [45]–[47]). *Xylt1* expression increased during the course of chondrogenic differentiation *in vitro* [48], and high Xylt serum activity has been linked to osteoarthritis [49]. Our expression, biochemical, and mutational analyses argue strongly for a predominant role of Xylt1 in CSPG production, specifically in cartilage matrix, thus providing an *in vivo* context for decades of *in vitro* studies.

In contrast to Xylts, Fam20 molecules were only recently identified, and our studies reveal *in vivo* functions of Fam20b [30]. Knock-down and over-expression studies in cell lines indicated that mouse *Fam20c* could promote odontoblast differentiation from mesenchymal stem cells [50], although the molecular mechanism for this role of Fam20c remains unclear. Renewed focus on Fam20 molecules arose from the revelation that humans with a skeletal disease called Raine syndrome have mutations in *FAM20C* [26], [27]. Subsequently, the paralogous protein Fam20b was shown *in vitro* to phosphorylate xylose on a nascent glycosaminoglycan side chain (Figure 5G), which appears to increase the likelihood that disaccharide repeats will be added by Exostosins and Chondroitin synthases [23]. Our expression and biochemical data show that *fam20b* functions similar to *xylt1* in cartilage PG production *in vivo*, and our analyses of *xylt1*, *fam20b*, and *uxs1* zebrafish mutants highlight the importance of xylosylation in skeletal development. Furthermore, all of these *fam20b* and *xylt1* mutants are homozygous viable, providing the only current vertebrate models in which to study how Fam20 and Xylt molecules affect the variety of human disease-related physiological processes for which they have been implicated [24]–[27], [49]. In particular, we note that humans with Raine syndrome display similar facial dysmorphies and osteosclerosis as seen in *fam20b* mutant zebrafish, potentiating these fish as an informative model by which to understand the etiology of Raine syndrome.

The mutations in *fam20b* and *xylt1* reported here help identify functional domains of the enzymes these genes encode. While Fam20b domains previously have not been probed experimentally, the conservation of amino acid residues among Fam20 family members suggests functional sites [30]. Both *fam20b^b1125^* and *fam20b^b1127^* disrupt cysteine residues that are highly conserved among vertebrates and may play a role in Fam20b molecular structure. Future *in vitro* enzyme assays will confirm whether such mutations abolish Fam20b kinase activity. Functional analyses of Xylt1 domains [28], [33], [51] did not target residues affected in our mutants, so *xylt1^b1128^* and *xylt1^b1189^* will provide new insights into Xylt1 function. The replacement of serine with alanine in *xylt1^b1189^* may alter minimally protein biochemical properties. As we show, this serine residue is conserved in Xylt1's across vertebrates, suggesting that it might serve as a target for phosphorylation that ultimately impacts enzyme performance. The *xylt1^b1128^* mutation alters some amino acids and then truncates a domain that is associated with protein-protein interactions (88645 superfamily; www.ensembl.org); thus, this alteration might change substrate recognition or inhibit molecular interactions with other glycosyltransferases in the proposed multienzyme complex [22], [52]. Future experiments designed to investigate whether skeletal defects of PG synthesis mutants arose merely from quantitative reduction in PG levels or rather also qualitative differences in protein substrate recognition will provide unique insight into the functional roles of PGs *in vivo*.

Our biochemical analyses demonstrate that the mutations in *fam20b* and *xylt1* reported here do not abolish cartilage PG synthesis completely. Mutations in both *fam20b* and *xylt1* reduce cartilage PG synthesis to similar extents; *fam20b;xylt1* double mutants are quantitatively more severe than either mutant alone; and *uxs1* mutants, which represent a complete loss of zygotic xylose-dependent PG production [18], exhibit an even more dramatic reduction in cartilage PG synthesis than *fam20b;xylt1* double mutants. Therefore, the alleles reported here might not be null, and/or redundant genes partially mask their loss. Five total zebrafish *fam20* genes have been reported (*fam20a*, *fam20b*, *fam20c1*, *fam20c2*, and *fam20c3*; [30]), and zebrafish have two *xylt* genes (*xylt1*, orthologous to human *XYLT1*, and *xylt2*, orthologous to human *XYLT2*; http://uswest.ensembl.org/Danio_rerio/). Expression and functional analyses of the full set of zebrafish *fam20* and *xylt* genes would test for redundant activities that might compensate for the loss of *fam20b* and *xylt1*. We do not expect *xylt2* to compensate for a loss of *xylt1* function during zebrafish skeletogenesis, because mice deficient for *Xylt2* exhibit polycystic kidney disease, but do not have skeletal defects [53].

Our finding that mutations in genes encoding CSPG synthesis enzymes accelerate endochondral ossification reveals that CSPGs can negatively regulate skeletogenic timing. This finding suggests to us that CSPGs might serve as therapeutics for skeletal defects that result from precocious developmental timing, such as craniosynostoses [54], [55]. Since various mutants affecting HSPG synthesis do not have increased perichondral bone [14]–[16], the gain in perichondral bone we describe here must be a CSPG-specific effect. In addition to this qualitative difference between HSPGs and CSPGs, PG levels may be interpreted quantitatively by developing skeletal cells. Homozygous *uxs1* mutants demonstrate loss of Alcian blue staining in cartilage in the absence of zygotic CSPGs, but *uxs1* mutants have delayed, rather than accelerated, bone formation [17], [18]. These data suggest that intermediate levels of CSPGs are required for the unique osteogenic acceleration observed in *fam20b* and *xylt1* mutants. In fact, degradation of CSPGs may be a normal, required stage of endochondral ossification, for lighter Alcian blue staining is associated with more mature cartilage matrix (see Figure 5; [56], [57]). Another explanation for the lack of accelerated bone formation in *uxs1* mutants is that these fish fail to produce both CSPGs and HSPGs. The gain in bone formation that loss of CSPGs imparts during endochondral ossification may depend upon HSPG function. This assertion is not supported by our data, however, as *fam20b* mutants exhibit increased perichondral bone while suffering loss of HSPG production in chondrocytes.

Together, our data argue that defective cartilage PG synthesis alters expression of transcription factors that determine the rates of chondrocyte maturation, thus changing the timing of perichondral bone formation. Increased expression of *runx2b* in *fam20b* and *xylt1* mutant chondrocytes likely drives early expression of chondrocyte maturation markers [39]–[41], such as *ihh* co-orthologs, which induce early perichondral bone [7], [8], [38]. As such, *fam20b* mutants may provide a completely new etiology for Raine syndrome: defects in chondrocyte differentiation underlie the increased perichondral bone (i.e., osteosclerosis) and skeletal dysmorphies observed in these humans [26], [27]. Premature cellular hallmarks of chondrocyte hypertrophy, including cytoplasmic clearing and loss of rough ER, Golgi complex, and mitochondria, accompany molecular features of early chondrocyte maturation in *xylt1* mutants. This premature terminal differentiation is also accompanied by changes in cartilage ECM, which contains well-defined, but more tightly packed collagen fibrils, and that might result from failure of GAGs to properly incorporate into the matrix.

The general hypothesis emerging from our work that CSPGs negatively regulate chondrocyte maturation is consistent with a recent, detailed study of the chick *Aggrecan* mutant [21]. Future work will aim to decipher the mechanism by which mutations in the cartilage PG synthesis pathway impact the timing of chondrocyte differentiation. Recent studies, for example, highlight a novel role for CSPGs in modulating growth factor signaling in developing cartilage [21], [58]. Here, we demonstrate that mutations in the CSPG synthesis pathway can accelerate developmental timing, thus expanding the ontology of genes regulating the rate of skeletogenesis.

## Materials and Methods

### Zebrafish lines

All fish lines were maintained and embryos raised according to established protocols [59] with IACUC approval. We obtained the *b1125*, *b1127*, *b1128*, and *b1189* mutant alleles through mutagenesis with *N*-ethyl-*N*-nitrosourea (ENU) in an AB background [59]. *ihha^hu2131^* fish were obtained from P. Ingham.

### Histological stains

Embryos were fixed in 2% PFA in PBS for 1 hr., washed in 100 mM Tris pH 7.5/10 mM MgCl_2_ for 10 min., stained in 0.04% Alcian blue/10 mM MgCl_2_/70% EtOH pH 7.5 overnight, taken through graded EtOH series (80% EtOH/100 mM Tris pH 7.5/10 mM MgCl2; 50% EtOH/100 mM Tris pH 7.5; 25% EtOH/100 mM Tris pH 7.5), bleached in 3% H_2_O_2_/0.5% KOH for 10 min. with lids open, washed twice in 25% glycerol/0.1% KOH for 10 min. each, stained in 0.01% Alizarin red/25% glycerol/0.1% KOH pH 7.5 for 30 min., and de-stained with two washes of 50% glycerol/0.1% KOH. Larvae were also incubated in 0.003% Alizarin red in Embryo Medium to visualize live mineralized bone.

### Mapping, cloning, and genotyping PG mutants

All oligonucleotide sequences appear in Figure S4. Sequence alignments were made using MultAlin [60]. RAD mapping localized *b1127* to LG20 [29]. Subsequent simple sequence repeat (SSR) mapping identified a genetic interval of 0.2 cM between SSRs defined by primers A+B and C+D on scaffold 2914 of Zv7 (http://uswest.ensembl.org/Danio_rerio/Info/Index) containing the *b1127* mutation (Figure 2A). In this interval, the SSR marker z20582 showed zero cross-overs with the mutant phenotype in 862 meioses. RNA isolated (TRI Reagent; Ambion Inc.) from live 5 dpf Alizarin red-stained zebrafish larvae that were screened for increased perichondral bone were made into cDNAs (First Strand Synthesis kit; Invitrogen Corp.). Full sequence of *fam20b* cDNA was determined by overlapping PCR fragments, generated from primers I+J and K+L. Four genes (*angptl1*, *ralgps2*, *blactl*, and *fam20b*) around z20582 were sequenced, and of these, mutant-specific coding sequence changes were identified only in the sixth coding exon of *fam20b* (Figure 2B, data not shown). PCR-based genotyping assays showed perfect correspondence between the *fam20b^b1127^* skeletal phenotype and this mutation (0 cross-overs/614 meioses; Figure 2B). The second allele in the *fam20b* complementation group, *b1125*, also mapped tightly to *fam20b*. The map cross contained three cross-overs between *fam20b^b1125^* and z20582 among 602 meioses (0.5 cM). *b1127* was genotyped by digesting PCR product from primers V+W with *HaeII*, which only cuts mutant sequence. *b1125* was genotyped with the marker z10805.

RAD mapping localized *b1128* to LG3 [29]. Subsequent SSR mapping defined a genetic interval of 0.7 cM between SSRs defined by primers E+F and G+H on scaffold 380 of Zv7 containing the mutation (Figure 2D). We focused on a predicted gene (LOC560951; www.ensembl.org) within this interval with homology to *xylosyltransferase1* (*xylt1*). Comparison of the Ensembl-predicted protein to Xylt1 of other vertebrates suggested that the annotated version of Xylt1 for zebrafish was lacking about 50 amino acids at the N-terminal portion of the protein. Therefore, we used 5′RACE on a 3 dpf cDNA library, using the nested primers M and N, along with universal primers (Clontech Laboratories, Inc.), to reveal an unannotated *xylt1* exon in zebrafish over 50 kb upstream of the annotated version. PCR and sequencing analyses confirmed the gene structure of zebrafish *xylt1*, which consists of 11 exons, similar to human *XYLT1* (Figure 2E). Full sequence of *xylt1* cDNA generated from primers O+N, P+Q, R+S, and T+U was submitted to GenBank (Accession: HQ692884). Sequencing of *xylt1* from *b1128* cDNA and gDNA revealed a splice donor mutation (G2103A) in exon 9 (Figure 2E). PCR-based genotype assays showed perfect correspondence between the *xylt1^b1128^* phenotype and this splice site mutation (0 cross-overs/738 meioses; Figure 2E). Sequencing from 5 dpf *xylt1^b1128^* mutant cDNAs showed that in the absence of the wild-type splice donor site, cryptic sites were used (6/6 clones). Although the most common of these (5/6 clones) resulted in a tetranucleotide insertion (Figure 2E, 2F), all mutant cDNAs would produce a frameshifted and truncated C-terminal portion of the zebrafish Xylt1 protein from at least amino acid 702 (out of 919). The second allele in the *xylt1* complementation group, *b1189*, also had a unique mutation in *xylt1*. cDNA sequencing revealed a T1600G mutation in the seventh coding exon of *xylt1^b1189^*, which altered highly-conserved amino acid 534 from Ser to Ala (Figure 2E, 2F). PCR-based genotype assays showed perfect correspondence between this mutation and the *xylt1^b1189^* phenotype (0 cross-overs/632 meioses; Figure 2E). *b1128* was genotyped by digesting PCR product from primers X+Y with *DdeI*, which only cuts wild-type sequence. *b1189* was genotyped by digesting PCR product from primers Z+AA with *HhaI*, which only cuts mutant sequence.

### *fam20b* rescue

Full-length *fam20b* was amplified from mutant and wild-type cDNAs with primers BB+CC and inserted into pDONR211 by BP recombination (Invitrogen Corp.). LR recombination reactions inserted *fam20b* under the control of *beta-actin2* or *hsp70l* promoters with GFP fused in-frame in the destination vector pDestTol2CG2, which also contained *cmlc2-GFP* [32]. mRNA from pCS2FA-transposase (http://chien.neuro.utah.edu/tol2kitwiki) was made (mMessage mMachine; Ambion Inc.). Approximately 3 nl of 100 ng/µl of *fam20b* expression plasmid, 70 ng/µl *transposase* RNA, and 0.2% phenol red were injected into one to four-cell embryos. Embryos containing green hearts were screened, and for heat shock activation, 55 hpf embryos were incubated at 40C for 20 min.

### Expression analyses

Whole-mount and section RNA *in situ* hybridization were carried out as described [61], [62]. Probes used were *runx2b*, *ihha*, *ihhb*, *ptch1*, *ptch2*, *col10a1* [18]. Nomenclature for *ptch* genes reflects a recent update, whereby previously termed *ptc1* is now *ptch2* and *ptc2* is now *ptch1* (www.zfin.org). Probes for *fam20b* and *xylt1* were created by cloning PCR amplicons from primers K+L and R+S, respectively, into pCR4-TOPO (Invitrogen Corp), digesting with *NotI*, and transcribing with T3 Polymerase. Primers DD+L and P+EE were used for *fam20b* and *xylt1* RT-PCR analyses, respectively. Immunostaining was carried out as previously reported [18].

### Alcian blue quantitation

Since Alcian blue binds sulfated glycosaminoglycans (GAGs; [63]), defects in cartilage PG production should be reflected by Alcian blue levels. As an indication of the levels of sulfated glycosaminoglycans in cell and tissue lysates, we modified the protocol of Bjornsson et al. (1998) as follows: larvae underwent all the stages except the Alizarin red staining of the Alcian blue, Alizarin red procedure outlined above, and tails were removed for genotyping. Samples were lysed (DNeasy kit; QIAGEN Inc.), spun briefly, and read spectrometrically at 620 nm. All analyses were performed in triplicate. Error bars represent standard error of the means.

### HPLC analyses

Glycosaminoglycan (GAG) biochemistry was carried out by the UCSD Glycotechnology Core Resource, according to protocols Pre006, Mis002, Mod004, Pro006, and Pro007 (http://glycotech.ucsd.edu/desc.html). Some disaccharide species did not appear in the majority of samples, due to their overall low levels, and were not included in analyses.

### Quantitation of perichondral bone

Digital images of flat-mounted, ventral views of ceratohyals in live Alizarin red-stained 6 dpf larvae were obtained under equivalent microscope settings, and subsequently analyzed with ImageJ software (http://rsbweb.nih.gov/ij/). Standard threshold values were applied to each image to define mineralized portions of ceratohyal perichondria. From eight samples each for genotyped mutants and wild types, mineralized area from the two ceratohyals was determined by the Analyze Particles function. Error bars represent standard error of the means.

### OPT imaging

Fish were fixed overnight in 4% PFA, washed for an hour in 1% KOH, bleached in 3% H_2_O_2_/0.5% KOH for 40 min. with lids open, washed in 1% KOH, stained overnight in 0.003% Alizarin red in 1% KOH, and de-stained in 1% KOH. After eyes were removed, heads were embedded in agarose, washed twice in methanol, and cleared in benzyl alcohol∶benzyl benzoate (2∶1). Images were captured using Bioptonics OPT Scanner 3001 M (MRC Technology).

### Transmission electron microscopy

Samples were processed as previously described [42], with the following modification. Zebrafish embryos were fixed in 2.5% glutaraldehyde in 0.1 M sodium cacodylate at room temperature for 1 hr and then at 4°C for at least 24 hr. They were then rinsed in 0.1 M sodium cacodylate and post-fixed with 1% osmium tetroxide in 0.1 M sodium cacodylate for 1 hr. Following additional rinsing, specimens were dehydrated step-wise in ethanol and then propylene oxide, infiltrated with resin step-wise, and then embedded in resin for 48 hr at 60°C. Ultra-thin 50 nm sections were collected on a Leica Ultracut Microtome and analyzed on a Phillips CM-12 Transmission Electron Microscope provided by VUMC Cell Imaging Shared Resource.

## Supporting Information

Figure S1 Exogenous wild-type *fam20b* expression does not alter skeletal phenotypes of wild types or *xylt1^b1128^* mutants, but can rescue cartilage of *fam20b^b1125^* mutants with a single induction at 55 hpf. A–E, dissected, flat-mounted pharyngeal skeletons of Alcian blue/Alizarin red-stained (A,B) and Alcian blue-stained (C–E) larvae. Injection of larvae with wild-type *fam20b* under control of the *beta-actin2* promoter did not alter skeletal phenotypes of wild types (A) or *xylt1^b1128^* mutants (B). Injection of larvae with wild-type *fam20b* under control of the *hsp70l* promoter rescued cartilage matrix production in *fam20b^b1125^* mutants when heat shocked at 55 hpf (E), compared to injected *fam20b^b1125^* embryos that did not undergo heat shock (D), while heat shock had no effect on uninjected control larvae (C). Abbreviations: HS = heat shock; UIC = uninjected control.
(TIF)

Figure S2 Cartilage proteoglycan secretion initiates at the same time in *xylt1* mutants and wild types. Alcian blue matrix was not apparent in pharyngeal arch 1 or 2 of wild types (A) and *xylt1^b1128^* mutants (B) at 48 hpf. Staining in the trabeculae of the neurocranium is evident in both wild types and *xylt1^b1128^* mutants. Alcian blue staining was obvious in pharyngeal arches of both wild types (C) and *xylt1^b1128^* mutants (D) at 60 hpf. Abbreviations: ch = ceratohyal; pa1 = pharyngeal arch1; pa2 = pharyngeal arch 2; pq = palatoquadrate; t = trabeculae.
(TIF)

Figure S3 Loss of proteoglycans in *fam20b*, *xylt1*, and *uxs1* mutants. A–C, HPLC quantitation of disaccharides in proteoglycans from whole larval lysates of *fam20b^b1127^*, *xylt1^b1128^*, and *uxs1* mutants at 5 dpf. A, Chondroitin sulfate levels were reduced in all mutants compared to wild-type siblings. B, Heparan sulfate levels were unaffected largely in *xylt1* mutants, but were decreased in *fam20b* and *uxs1* mutants, compared to wild-type siblings. C, Instead of plotting values as a percentage of wild-type, this graph illustrates picomoles of each disaccharide species in *fam20b^b1127^* and their wild-type siblings, demonstrating that the relatively high level of UA-2S-GlcNAc in *fam20b^b1127^* mutants (* in B) is due to the low levels of this disaccharide that typically were detectable in these samples (* in C).
(TIF)

Figure S4 Oligonucleotide sequences used in this study.
(DOC)
