# Supplementary figures and images for: Mutations in fam20b and xylt1 Reveal That Cartilage Matrix Controls Timing of Endochondral Ossification by Inhibiting Chondrocyte Maturation
Source: PLoS Genet. 2011 Aug 25;7(8):e1002246. doi: 10.1371/journal.pgen.1002246 (PMC3161922; doi:10.1371/journal.pgen.1002246)

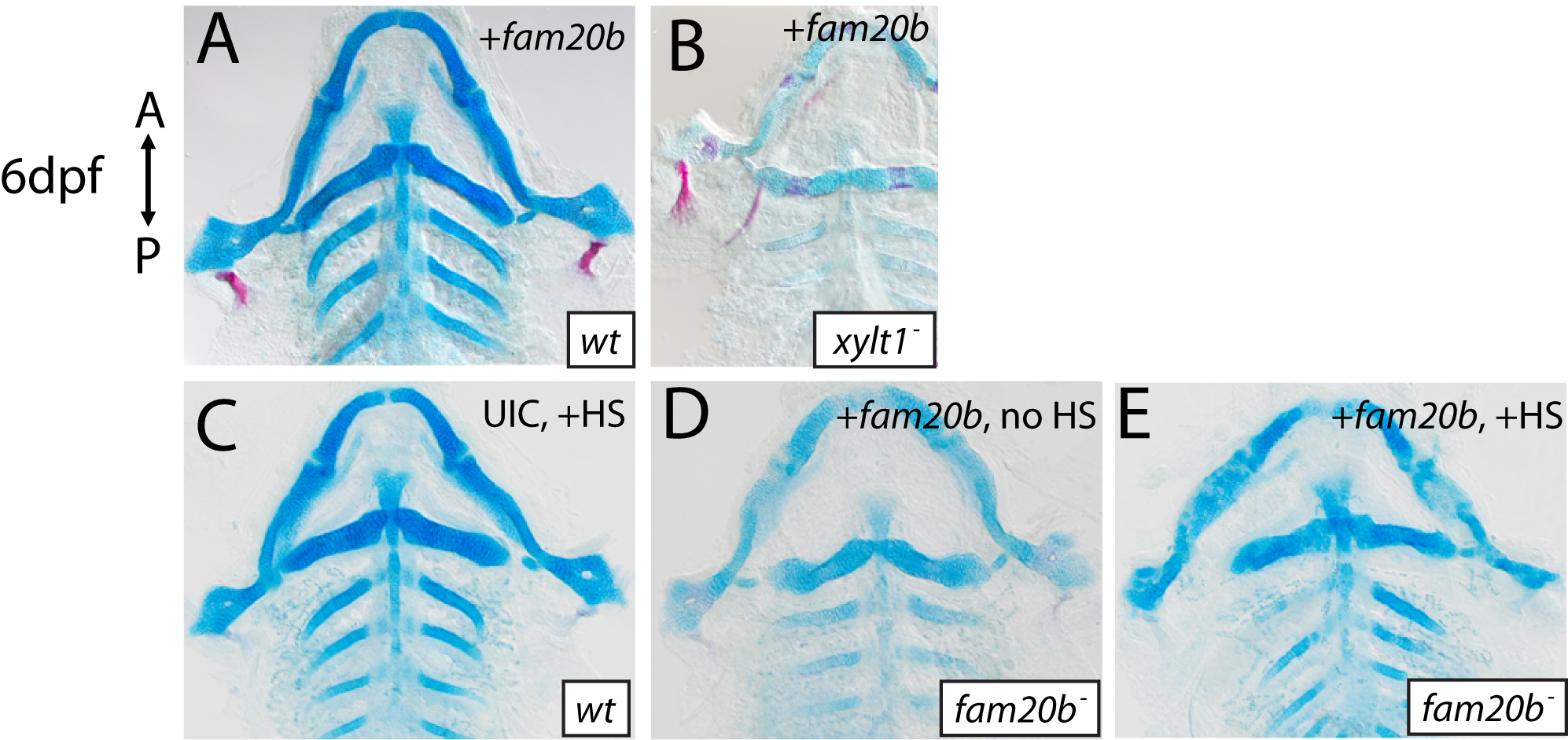

Supplement: Figure S1 — Exogenous wild-type fam20b expression does not alter skeletal phenotypes of wild types or xylt1b1128 mutants, but can rescue cartilage of fam20bb1125 mutants with a single induction at 55 hpf. A–E, dissected, flat-mounted pharyngeal skeletons of Alcian blue/Alizarin red-stained (A,B) and Alcian blue-stained (C–E) larvae. Injection of larvae with wild-type fam20b under control of the beta-actin2 promoter did not alter skeletal phenotypes of wild types (A) or xylt1b1128 mutants (B). Injection of larvae with wild-type fam20b under control of the hsp70l promoter rescued cartilage matrix production in fam20bb1125 mutants when heat shocked at 55 hpf (E), compared to injected fam20bb1125 embryos that did not undergo heat shock (D), while heat shock had no effect on uninjected control larvae (C). Abbreviations: HS = heat shock; UIC = uninjected control. (TIF) [file pgen.1002246.s001.tif]

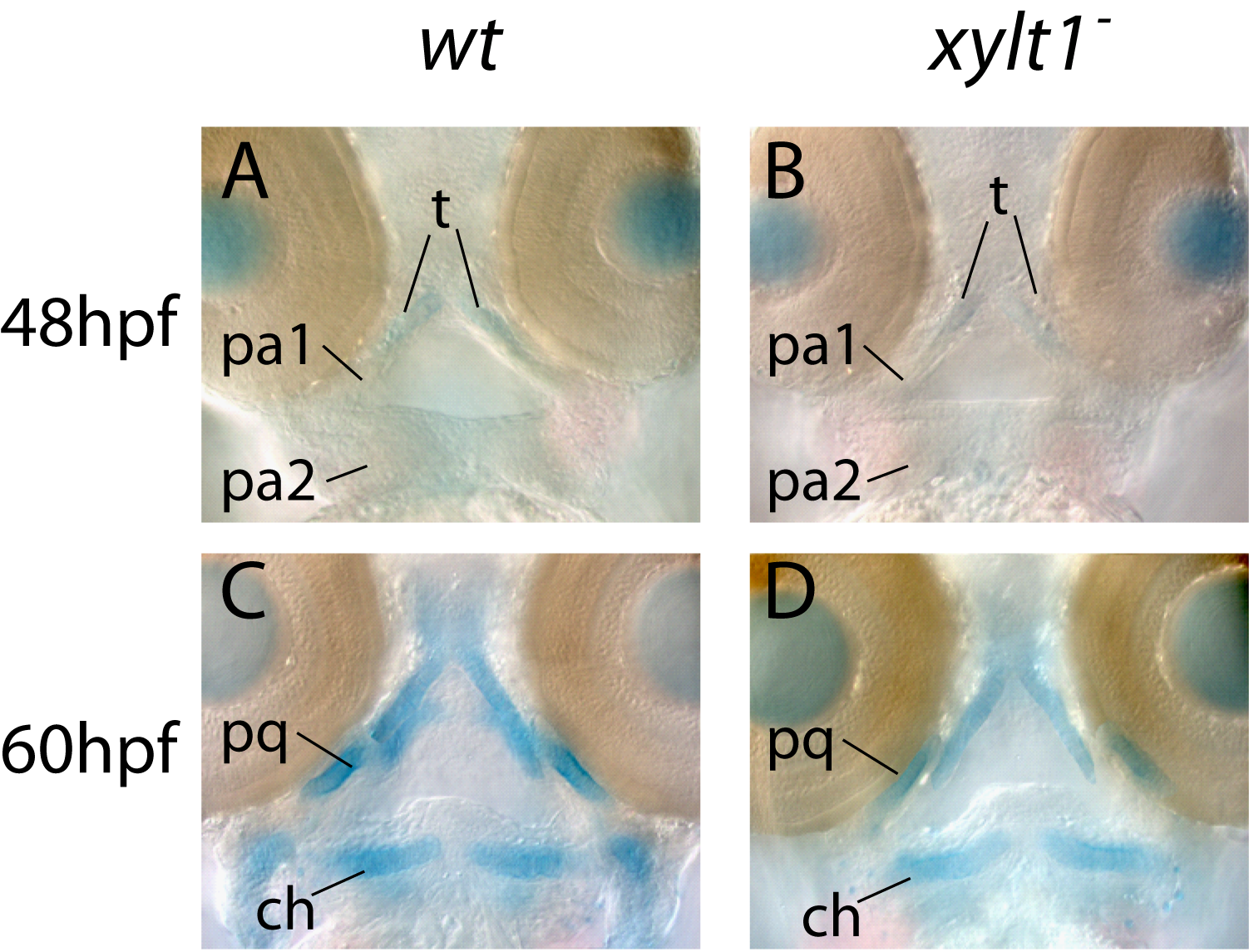

Supplement: Figure S2 — Cartilage proteoglycan secretion initiates at the same time in xylt1 mutants and wild types. Alcian blue matrix was not apparent in pharyngeal arch 1 or 2 of wild types (A) and xylt1b1128 mutants (B) at 48 hpf. Staining in the trabeculae of the neurocranium is evident in both wild types and xylt1b1128 mutants. Alcian blue staining was obvious in pharyngeal arches of both wild types (C) and xylt1b1128 mutants (D) at 60 hpf. Abbreviations: ch = ceratohyal; pa1 = pharyngeal arch1; pa2 = pharyngeal arch 2; pq = palatoquadrate; t = trabeculae. (TIF) [file pgen.1002246.s002.tif]

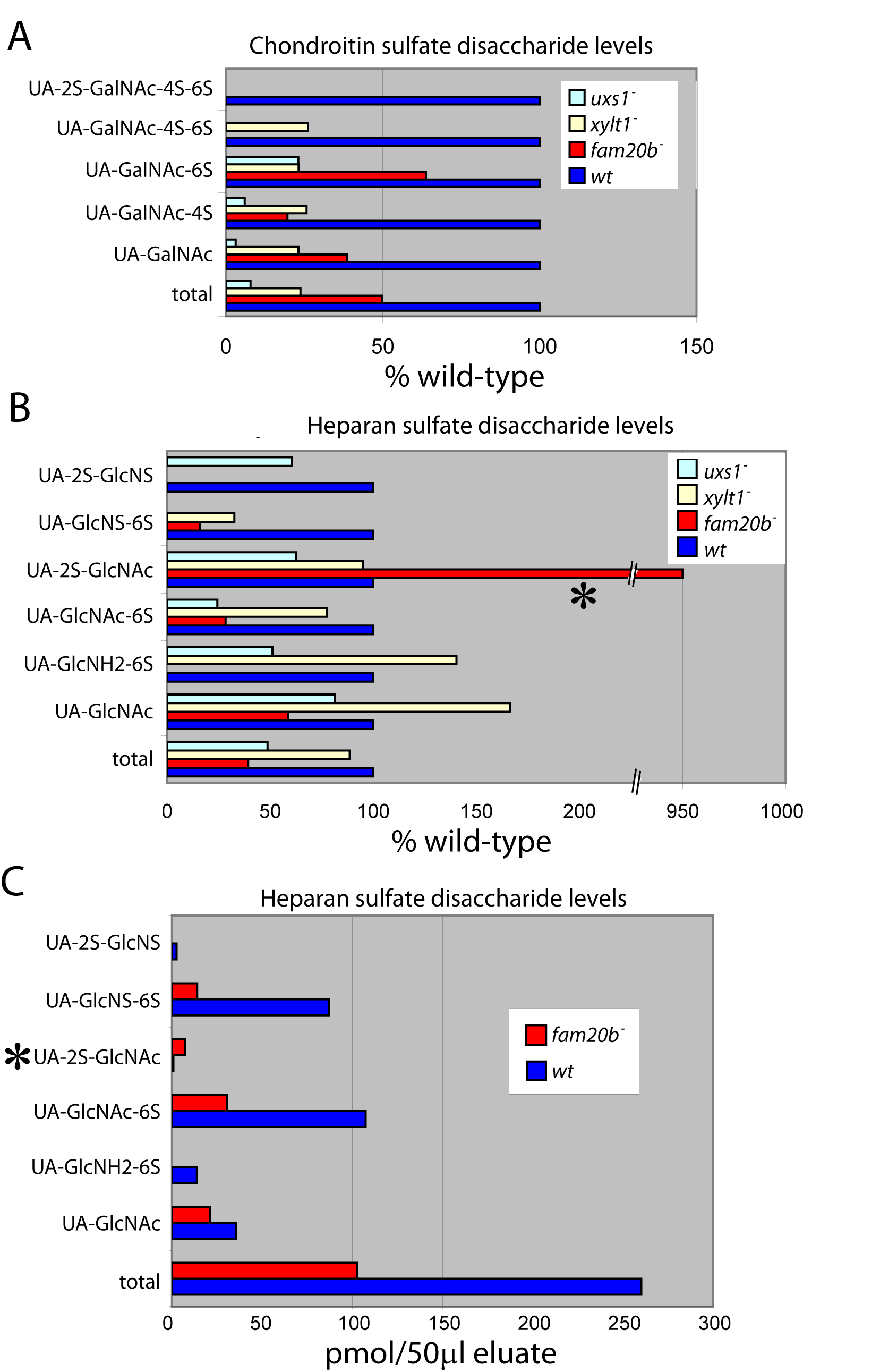

Supplement: Figure S3 — Loss of proteoglycans in fam20b, xylt1, and uxs1 mutants. A–C, HPLC quantitation of disaccharides in proteoglycans from whole larval lysates of fam20bb1127, xylt1b1128, and uxs1 mutants at 5 dpf. A, Chondroitin sulfate levels were reduced in all mutants compared to wild-type siblings. B, Heparan sulfate levels were unaffected largely in xylt1 mutants, but were decreased in fam20b and uxs1 mutants, compared to wild-type siblings. C, Instead of plotting values as a percentage of wild-type, this graph illustrates picomoles of each disaccharide species in fam20bb1127 and their wild-type siblings, demonstrating that the relatively high level of UA-2S-GlcNAc in fam20bb1127 mutants (* in B) is due to the low levels of this disaccharide that typically were detectable in these samples (* in C). (TIF) [file pgen.1002246.s003.tif]
