# Supplementary material for: Mutations in fam20b and xylt1 Reveal That Cartilage Matrix Controls Timing of Endochondral Ossification by Inhibiting Chondrocyte Maturation
Source: PLoS Genet. 2011 Aug 25;7(8):e1002246. doi: 10.1371/journal.pgen.1002246 (PMC3161922; doi:10.1371/journal.pgen.1002246)
Supplement: Figure S4 — Oligonucleotide sequences used in this study. (DOC) [file pgen.1002246.s004.doc]

Eames *et al.* Supplementary Figure 4

Oligonucleotide sequences

In the order listed in Methods:

A=2914.3f (5’-TTGCACCCACTGAGAGTGTT)

B=2914.3r (5’-GGATGGGTTTGTGCCATTAT)

C=2914.6f (5’-GCCTCTGTTCACTGCTAGGG)

D=2914.6r (5’-ATGTAGGGCCAAAGGAGGAC)

E=380.2f (5’-GACGCTATCAGATGCAGCAA)

F=380.2r (5’-CCCAGAAAACTCCACTCATTC)

G=380.8f (5’-GGAAATCTGTGCGTGTCTGA)

H=380.8r (5’-ACTCATTTCCAGCAGGAGGA)

I=fam20b.1f (5’-CACCAGGCTGAACTCCAAAT)

J=fam20b.1r (5’-GTTTCTGTAAGGGCCAGACG)

K=fam20b.2f (5’-TGAGCACTTTCCTGATGCAC)

L=fam20b.2r (5’-CTCGGCGCTGAATAAATCC)

M=xt1.5race.r3 (5’-GCTGTCCACACGGACTTTCTCCTTGG)

N=xt1.5race.r4 (5’-TGTGTTTGGGAACGTGCACCGAAGT)

O=glytra.1ef (5’-GAGCCACAAAGCGGACTGT)

P=glytra.1cf (5’-CCGGCCCAAGGAGAAAGT)

Q=glytra.1r (5’-GAATCGAGCGTTGTCTCTCC)

R=glytra.2f (5’-GGCTCTCGCTCACCAGTATC)

S=glytra.2r (5’-AGGTCTCCAGGGTTTCCAGT)

T=glytra.3f (5’-CGACGCACATCTCACTCACT)

U=glytra.3r (5’-TCATGTCTTGCGCTTGAGAG)

V=f20bex8f (5’-AAACTCCACGGTTTTGGTTTG)

W=f20bex8r (5’-GCAAAAACACACCGCTTTTT)

X=xt1ex8f2 (5’-ACTGGAAACCCTGGAGACCT)

Y=xt1ex8r2 (5’-AAATGGCCAACAGAGGACAC)

Z=1189geno.f1 (5’-GAAAGCTGGGTTGCAAGTGT)

AA=1189geno.r1 (5’-TCAGCTCTGCCACTGAAAAG)

BB=f20b.attB1.f2 (5’-GGGGACAAGTTTGTACAAAAAAGCAGGCTAGTAGCGCAAGGGAGATCAA)

CC=f20b.attB2.r1 (5’-GGGGACCACTTTGTACAAGAAAGCTGGGTYCGGATGAGGCAGGTTCAT)

DD=f20bex9f (5’-GTCCACCTGGAACAGACTGAA)

EE=xt1ex1r1 (5’-ACATTTGGGTGGTTGGTCAT)
